# S-nitrosylation of E3 ubiquitin-protein ligase RNF213 alters non-canonical Wnt/Ca+2 signaling in the P301S mouse model of tauopathy

**DOI:** 10.1038/s41398-019-0388-7

**Published:** 2019-01-29

**Authors:** Haitham Amal, Guanyu Gong, Elizabeta Gjoneska, Sarah M. Lewis, John S. Wishnok, Li-Huei Tsai, Steven R. Tannenbaum

**Affiliations:** 1 Department of Biological Engineering, Massachusetts Institute of Technology, Cambridge, MA 02139 USA; 2 The Picower Institute for Learning and Memory, Department of Brain and Cognitive Sciences, Massachusetts Institute of Technology, Cambridge, MA 02139 USA; 3 Department of Chemistry, Massachusetts Institute of Technology, Cambridge, MA 02139 USA

## Abstract

Mutations in the *MAPT* gene, which encodes the tau protein, are associated with several neurodegenerative diseases, including frontotemporal dementia (FTD), dementia with epilepsy, and other types of dementia. The missense mutation in the *Mapt* gene in the P301S mouse model of FTD results in impaired synaptic function and microgliosis at three months of age, which are the earliest manifestations of disease. Here, we examined changes in the S-nitrosoproteome in 2-month-old transgenic P301S mice in order to detect molecular events corresponding to early stages of disease progression. S-nitrosylated (SNO) proteins were identified in two brain regions, cortex and hippocampus, in P301S and Wild Type (WT) littermate control mice. We found major changes in the S-nitrosoproteome between the groups in both regions. Several pathways converged to show that calcium regulation and non-canonical Wnt signaling are affected using GO and pathway analysis. Significant increase in 3-nitrotyrosine was found in the CA1 and entorhinal cortex regions, which indicates an elevation of oxidative stress and nitric oxide formation. There was evidence of increased Non-Canonical Wnt/Ca++ (NC-WCa) signaling in the cortex of the P301S mice; including increases in phosphorylated CaMKII, and S-nitrosylation of E3 ubiquitin-protein ligase RNF213 (RNF-213) leading to increased levels of nuclear factor of activated T-cells 1 (NFAT-1) and FILAMIN-A, which further amplify the NC-WCa and contribute to the pathology. These findings implicate activation of the NC-WCa pathway in tauopathy and provide novel insights into the contribution of S-nitrosylation to NC-WCa activation, and offer new potential drug targets for treatment of tauopathies.

## Introduction

Tau protein is associated with several neurodegenerative diseases, including Alzheimer’s disease (AD), and different frontotemporal dementias, as well as dementia following traumatic brain injury^1^. Tau is a member of the microtubule-associated proteins (MAPs) that is located on chromosome 17q21.31 in humans and coded by the *MAPT* gene^2^.

Tau’s main function is to promote microtubule (MT) assembly and modulate the stability of axonal MTs^3,4^. Tau is a phosphoprotein and is known to be phosphorylated on Serine and Threonine sites^5^. Tau phosphorylation sites are clustered in regions flanking the MT binding repeats and hyperphosphorylation of tau inhibits MT assembly^6,7^ leading to the formation of distinct aggregates of tau^1^, which constitute neurofibrillary tangles (NFTs) in AD^8^. The main dogma in the field is that filamentous tau aggregates are the most destructive and pernicious forms of tau^9^. Tau also has a major role in axonogenesis, neurite outgrowth^10^, and modulation of the interaction of MTs and actin polymers^11^.

Tau also acts as a scaffold protein that interacts through its amino-terminal projection domain with the Src family tyrosine kinase Fyn^12^, which phosphorylates the NMDAR subunit 2 (NMDAR2). Phosphorylation of NMDAR by Fyn, facilitates the interaction of NMDAR with PSD-95^13,14^, leading to NMDAR activation, Ca++ influx, and synaptic excitotoxic downstream signaling^15^. This is important because PSD-95 interacts with neuronal nitric oxide synthase (nNOS) that mediates synaptic association and activation of nNOS^16^.

S-nitrosylation, the NO-mediated post-translational modification of cysteine thiols (SNO), is known to be involved in different neuropathology, such as AD^17–19^, Parkinson’s^20^ and Huntington’s disease^18,19^, and other neurodegenerative disorders^21–23^. Recently and for the first time in the literature, we showed S-nitrosylation involvement in autism spectrum disorder mouse model^24^. SNO regulates the localization and activity of many key enzymes and receptors^18,25,26^ leading to modulation of signaling pathways, synaptic plasticity, axonal elongation, movement of proteins to the cell membrane, and protein assembly^18,25^.

We previously profiled S-nitrosylation in the CK-p25 mouse model of AD, which exhibits DNA damage, aberrant gene expression, increased amyloid-β levels, and neuronal and synaptic loss followed by cognitive impairment and tau hyperphosphrylation and aggregation at later stages^27^. Our work on the CK-p25 mouse model showed that there was increased S-nitrosylation of proteins important for synapse function, and metabolism, and correlated with amyloid formation^17^. In the current study we profiled the changes in S-nitrosylation in the P301S tau transgenic (Tg) mouse model which overexpresses the human tau mutation identified in early onset familial FTD^28^. The P301S mice exhibit NFTs in the cortex, hippocampus and amygdala and impairment in spatial learning and memory at six months of age^29^. They also showed impaired synaptic function and microgliosis at three months of age, which are the earliest pathological manifestations in this model^29^. We tested whether there is a convergence of pathways between the CK-p25 mouse that models changes in enzymatic activity in AD, and P301S mouse that models genetic change in FTD^28,30^.

To detect molecular changes that take place during early stages of disease progression in the P301S model, we tested the mice at two months of age. We used the SNOTRAP approach developed in our laboratories^17,31^ to detect differentially S-nitrosylated proteins in P301S mice and littermate age-matched controls. This was followed by GO and pathway analysis that aggregates observations across multiple proteins to identify commonality in the affected biology. This approach allowed us to identify and characterize key proteins, biological processes, and important signaling pathways that could serve as novel therapeutic targets for treatment of tauopathies.

We identified proteins in the NC-WCa signaling pathway that were differentially S-nitrosylated in the P301S mouse model and, as a consequence, the pathway was over-activated in the cortex of these mice.

The major role of the NC-WCa is to regulate calcium release from the endoplasmic reticulum (ER) and stabilize the calcium levels in the cell^32,33^. The non-canonical Wnt signaling pathway is a series of molecular signals initiated by binding of a WNT protein to the frizzled family receptor on the surface of the target cell, followed by propagation of the signal via effectors other than beta-catenin^32^. This leads to downstream changes such as phospholipase C (PLC) activation^34^. PLC activation leads to IP3 production which binds to its receptor on the ER resulting in calcium release^32^ and downstream activation of kinases, including calmodulin-dependent protein kinase II (CaMKII). Ca++ release also leads to CaMKII activation and autophosphorylation^35^ and subsequent phosphorylation of NMDAR, which further potentiates Ca++ influx^32,36^ and NC-WCa activation.

The role of Wnt signaling pathways in AD mainly as a neuro-protective mechanism against amyloid beta plaques has previously been investigated, however, the function of the NC-WCa pathway in tauopathy has not yet been examined^32,36,37^. Here we identify key proteins that are potential indicators of early disease manifestation and show for a first time that S-nitrosylation is a mechanism that regulates the function of the NC-WCa pathway, which is disrupted in a tauopathy model of neurodegeneration.

## Materials and methods

### Animals

All mouse experiments were approved by the Committee on Animal Care of the Division of Comparative Medicine at Massachusetts Institute of Technology (MIT). Two-month-old male tau P301S mice and respective wild type (WT) control mice were used. For tissue dissection, mice were chosen in an alternate order, and similarly, samples preparation was done in an alternate order.

Cortex and hippocampus were collected on ice, immediately flash frozen in liquid nitrogen and stored at −80 °C.

### Materials and reagents

Biotin-PEG3-propionic acid was purchased from ChemPep Inc. Protease cocktail inhibitors were purchased from Sigma-Aldrich and sequencing-grade modified trypsin from Promega. High–performance liquid chromatography (HPLC) and the liquid chromotography–mass spectrometry solvents were HPLC grade. Acetonitrile (ACN) and distilled water for MS use were purchased from Sigma-Aldrich. Vivapsin 10 k molecular weight cut-off (MWCO) filters were purchased from Sartorius Stedim NA. Synthesis of SNOTRAP-biotin and NMR analysis are described in detail by Seneviratne et al.^17^. All sample preparation was conducted in dark conditions at room temperature (RT).

### Mass spectrometry (MS) sample preparation

Tissues were homogenized in a 1 ml (for one intact cortex) or 0.5 ml (for one intact hippocampus) of lysis buffer on ice using a Teflon pestle and a Jumbo Stirrer (Thermo Fisher). The lysis buffer was freshly prepared and contained 250 mM HEPES-NaOH (pH 7.7), 1 mM EDTA, 0.1 mM neocuproine, 8 M urea, 20 mM

Iodoacetamide (IAM), and 1% protease inhibitor cocktail (Sigma-Aldrich, Cat. No. P8340).

The homogenates were centrifuged at 12000×*g* for 10 min at 4 °C and supernatants were collected. The protein concentration was determined by the Bradford assay (Bio-Rad, Cat. No. 500-0006). Equal amounts of cortical protein (4 mg) from three mice were pooled into one cortex sample; equal amounts of cortex protein (2 mg) from six mice were pooled as the negative sample. After mixing, there were two cortex samples and one negative cortex sample for P301S Tg and WT mice, respectively. In the case of the hippocampus, we pooled 1 mg protein from three mice as one hippocampus sample and 0.5 mg protein from six mice as the hippocampus negative sample.

Negative controls were generated by treatment with 10 mM TCEP for 30 min at 37 °C after sample mixing. Samples were then alkylated with 30 mM IAM in the presence of 2.5% SDS in the dark at 37 °C. After alkylation, samples were washed with three volumes of 8 M urea (in 50 mM HEPES, pH 7.7) twice and 50 mM HEPES (pH 7.7) once followed by centrifugation at 5000×*g* for 30 min at 4 °C with 10 K MWCO spin filters (pre-rinsed with water once, Sartorius Corporation, Cat. No. VS15T01).

SNOTRAP labeling stock solutions (in 40% acetonitrile) were added to all samples to reach a final concentration of 1.25 mM (in 50 mM HEPES buffer at pH 7.7) to selectively convert SNO to stable disulfide-iminophosphorane. Samples were incubated with SNOTRAP solution at room temperature for 1.5 h. After SNOTRAP labeling, excessive reagents were removed by three washes with 50 mM HEPES, pH 7.7 buffer with 10 K filters.

After ultrafiltration, 200 μl of pre-rinsed Streptavidin agarose beads (Pierce, Cat. No. 20349) were added to each sample and incubated for 1 h at room temperature with gentle agitation. The beads were washed with washing buffer 1 (50 mM HEPES, 150 mM NaCl, 0.1 % SDS, pH 7.7) three times and then with washing buffer 2 (50 mM HEPES, pH 7.7) three times. Protein was eluted with 10 mM TCEP (in 50 mM HEPES, pH 7.7) and then alkylated with 10 mM IAM. After alkylation, samples were trypsinized (Promega, Cat. No. V5111) at 37 °C for 4 h and then desalted with C18 StageTips.

### MS analysis

Peptides were analyzed using the Agilent HPLC-Chip/MS system, consisting of a micro-autosampler, a capillary and nano flow pump, and the Chip-Cube that interfaces LC modules and the MS instrument. Water (0.1% Formic Acid (FA)) and ACN (0.1% FA) were used as mobile phases A and B, respectively. Peptide separations were carried out on a Polaris-HR-Chip-3C18 HPLC-Chip (Agilent Technologies, Cat. No. G4240-62030), consisting of a 360-nL enrichment column and a 75 μm × 150 mm analytical column, both of which were packed with Polaris C18-A, 180 A, 3-μm stationary phase. Peptides were loaded onto the enrichment column from the autosampler at a constant flow of 2 μl/min provided by the capillary pump. A 55-min gradient started at 3% B at 300 nl/min and increased to 30% B from 2 to 35 min, to 60% B at 40 min, to 90% B at 45 min and then was held for 5 min and followed by a 5 min post-run at 3% B. MS analysis was performed with an Agilent 6550 Accurate Mass Ion Funnel QTOF Chip-MS System operated in positive-ion mode. MS spectra were acquired in the 1700 Da extended dynamic range mode (2 GHz) using the following settings: ESI capillary voltage, 1960 V; fragmentor, 360 V; Octopole RF peak, 750 V; drying gas, 13 L/min; drying temperature, 225 °C. Data were acquired at a rate of 6 MS spectra per second and 3 MS/MS spectra per second in the mass range of *m/z* 300–1700 for MS and 50–1700 for MS/MS and stored in centroid mode. The maximum number of precursors per cycle was 20, with a threshold of 5000 ions in a precursor abundance-based scan speed in peptide isotope model, with +2, +3 and above charge state preference, and with active exclusion after 1 spectrum and released after 0.15 min. Fragmentation energy was applied at a slope of 3.1 V/100 Da with a 1.0 offset for doubly charged precursors, 3.6 V/100 Da with a −4.8 offset for triply and multiply charged precursors. Mass accuracy was maintained by using the internal reference ion m/z 1221.9906. Agilent MassHunter Workstation software was used for data acquisition. Two technical runs were conducted for each sample.

### MS data processing

Agilent Spectrum Mill MS proteomics Workbench B.05 was used for peak list generation, database searching, and FDR estimation. Parameters for data extractions were as follows: cysteine carbamidomethylation for fixed modification, precursor MH + 300–8000 Da, scan time range 0–200 min, sequence tag length > 1, merge scans with same precursor m/z ± 30 s ± 0.05 m/z, default for precursor charge, and to find 12 C precursor, and MS noise threshold 100 counts. MS/MS spectra were searched against the mouse SwissProt protein database with ±20 ppm precursor ion tolerance and ±50 ppm fragment ion tolerance. The search included variable modifications of methionine oxidation, protein N-terminal acetylation, deamidation of asparagine and fixed modification of cysteine carbamidomethylation. Peptide and protein False Discovery Rate (FDR) was set to 1.2% and Spectrum Peak Intensity was set to 30% for peptide and protein identification. The mass spectrometry proteomics data have been deposited to the ProteomeXchange Consortium (http://proteomecentral.proteomexchange.org) via the PRIDE partner repository with the dataset identifier <PXD010106>.”

### Bioinformatics and statistics

The lists of SNO-proteins were submitted to “MetaCore from Thomson Reuters”, MetaCore™ version 6.34 build 69200. GO processes, Pathway Maps, and Networks were generated. Terms that were below 0.05, following Benjamini corrected FDR, were considered significant. Pathway, GO, and networks figures were generated by Metacore. STRING^38^ was used to analyze the protein-protein interaction of SNO-proteins (http://string-db.org). Highly reliable interactions (score > 0.7) from neighborhood, gene fusion, co-occurrence, co-expression, experiments, databases and text mining lists were kept. The protein interaction network was then illustrated with Cytoscape 3.2.1. Sample sizes were chosen on the basis of preliminary experiments and our experience with similar experiments. Blind-coded experiments were done, in which the researchers who obtained the data were unaware of the specific genotype of mice.

### Western blot (WB)

WB was used to measure levels of proteins. Supernatant of the homogenized tissue was diluted with reducing buffer, Laemmli sample buffer (Biorad, 161–0737) and electrophoresed on Tris-HCL 4–20% pre-cast linear gradient gel (Biorad, 4561093) and transferred to PVDF membrane (Biorad, 1620174). The membranes were blocked with 5% milk in TBS-0.05% Tween 20 for 1 h at RT, incubated with primary antibody overnight at 4 °C and, after six washes with TBS-0.5%Tween 20, the membrane was incubated with a horseradish peroxidase-conjugated secondary antibody for 1 h at RT. Protein bands were visualized using ECL reagent. The bands were captured using Fluorchem^TM^ 8900 (Alpha Innotech). Primary antibodies were purchased as follow: nNOS (Cell signaling, 4231 S, 1/1000), iNOS (Santa Cruz, C11, 1/500), CaMKll (Cell Signalling Technology (CST), 3362, 1/1000 dilution), P-CaMKll (Thr286) (CST, 12716, 1/1000 dilution), GAPDH (CST, 21185, 1/1000 dilution), CREB (Invitrogen, MA1-083, 1/500 dilution), Phospho-CREB-Ser133-(Invitrogen, PA1-4619, 1/1000 dilution), NFAT1 (CST, D43B1, 1/1000 dilution), and FILAMIN-A (CST, 4762, 1/1000 dilution).

Secondary antibodies used were goat-anti-rabbit (Invitrogen, A11036, 1/10000 dilution) and goat-anti-mouse (Invitrogen, A11001, 1/10000 dilution).

Each group contained three biological replicates. Each biological replicate comprised three pooled cortex or hippocampus tissues from three different mice.

### Immunohistochemistry (IHC)

IHC staining of mouse brain sections was performed using the following antibodies: Nitrotyrosine antibody (Millipore, AB5411, 1/200), NeuN antibody (Millipore, MAB377, 1/200), GFAP antibody (Abcam, ab7260, 1/500), Iba1 (Abcam, ab15690, 1/500). Briefly, whole mouse brains were fixed in 10% formalin, cut into coronal slices and embedded in paraffin blocks. Formalin-fixed paraffin-embedded (FFPE) tissue sections were deparaffinized in 100% Xylene and gradient washed in 100, 90 and 70% ethanol and ddH2O. Sections were boiled in the antigen retrieval buffer (Dako, pH = 6.0, S1700) at 95 °C for 20 min. Sections were blocked with 3% BSA in PBS-Triton X100 (0.3% v/v) overnight, incubated with primary antibody, washed, and incubated with Secondary antibody: goat-anti-rabbit (Invitrogen, A11036, 1/500 dilution) and goat-anti-mouse (Invitrogen, A11001, 1/500 dilution) at RT for 1 h. Sections were washed and counterstained with 4’,6-diamidino-2-phenylindole (DAPI). Each group contained six mice, when each mouse represents one biological sample.

## Results

**Fig. 1:**
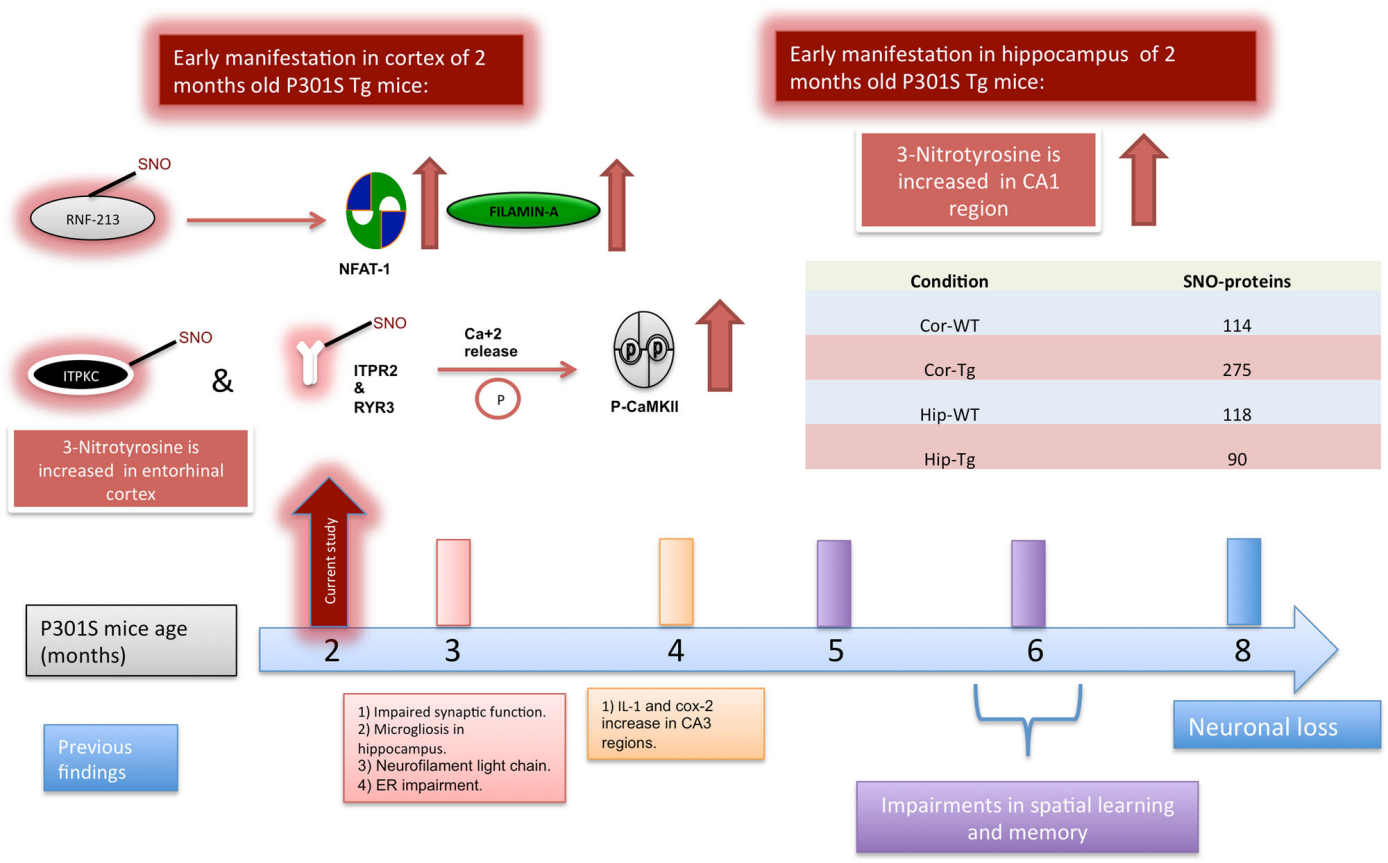
Timeline chart presenting pathology of P301S model from 3 to 8 months of age in the bottom. Top presents the findings of the current study in 2-month-old mice. The table presents the number of SNO-proteins identified in each group

To detect early molecular changes in the P301S Tg model, we profiled S-nitrosylation in two brain regions, the cortex and hippocampus, of P301S mice (Cor-Tg and Hip-Tg, respectively) and WT (Cor-WT and Hip-WT) controls, at two months of age (Fig. 1).

### The detection of proteins differentially S-nitrosylated in Tg *vs*. control mice

We identified differences in S-nitrosylation between the Tg and WT groups. A higher number of SNO-proteins were detected in Cor-Tg compared to Cor-WT, while there was no major difference in the number of SNO-proteins in the hippocampus (See Fig. 1 and Supplementary Figure 1A–D). In both regions, the identity of most of the proteins was different between WT and Tg. Protein IDs of the four different groups are presented in Supplementary Table 1. Analysis of the SNO-proteins according to function showed a higher number of enzymes, including kinases and transcription factors, that were S-nitrosylated in Cor-Tg compared to Cor-WT (see Supplementary Figure 2).

### Gene ontology and pathway analysis of the S-nitrosoproteome

**Fig. 2:**
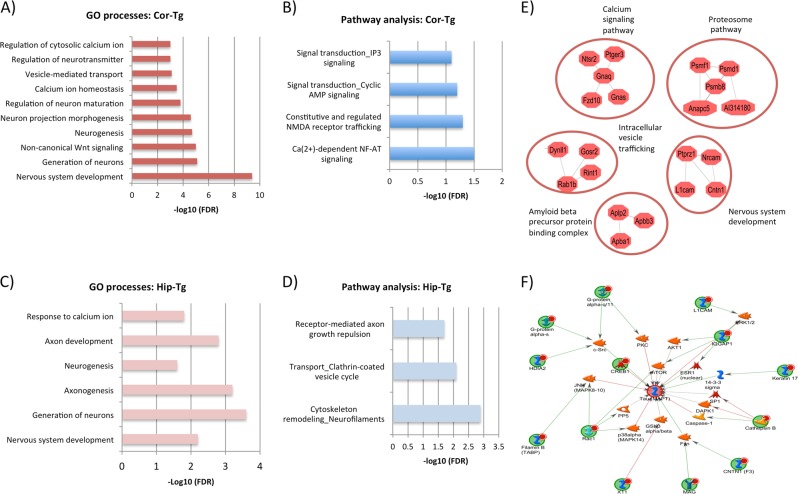
**a**, **b** Enrichment of GO processes and pathway analysis of the SNO-proteins that were found in the cotex of the P301S Tg mice (Cor-Tg). **c**, **d** Enrichment of GO processes and pathway analysis of the SNO-proteins that were found in the hippocampus of the P301S Tg mice (Hip-Tg). **e** Physical protein interaction analysis of the SNO-proteins that were found in Cor-Tg. **f** Network analysis of SNO-proteins of Cor-Tg with tau protein * Bars represent the –log10 of the Benjamini corrected false discovery rate (FDR)

Our aim in the downstream GO and pathway analysis is to aggregate observations across the SNO-proteins to enable the characterization and identification of (neuro) biological processes and signaling pathways that are affected in tauopathy. GO analysis revealed enrichment of proteins associated with neuronal and synaptic functions, such as nervous system development, neurogenesis, and vesicle-mediated transport, specifically in the cortex of the P301S Tg mouse, but not the WT control (Fig. 2a, Supplementary Table 2). Proteins associated with calcium-related functions such as regulation of calcium ions and calcium homeostasis, were also exclusively enriched in the cortex of the P301S Tg mice. Lastly, components of the non-canonical Wnt signaling pathway were enriched in Cor-Tg mice as well. Specifically we observed S-nitrosylation of the Rac family small Gtpase 1, Frizzled-10 (FZD10), and inositol 1,4,5-trisphosphate receptor type 2 (IP3R2). Consistent with the GO analysis, pathway analysis of Cor-Tg (Fig. 2b) showed enrichment of specific calcium-related pathways, such as signal transduction_IP3 signaling pathway (see Metacore pathway in Supplementary Figure 3) and NMDAR trafficking pathway (see Metacore pathway in Supplementary Figure 4).

GO and pathway analysis of Hip-Tg (Fig. 2c, d respectively and Supplementary Table 3) also showed enrichment of several neuronal and calcium-related processes and pathways, such as response to calcium ions, nervous system development and axon growth (see axon growth pathway by Metacore in Supplementary Figure 5).

These results suggest that neuronal, synaptic, and calcium processes are affected by differential S-nitrosylation in the P301S Tg mice.

### Network analysis of S-nitroso-proteome

Process network analysis was performed to assess overall functional character of the SNO-proteins set in each group. In particular, SNO-proteins in Cor-Tg mice that function in calcium and neuronal processes were clustered in networks enriched for: (1) Non-canonical Wnt signaling pathway (FDR = 4.682e-4) consisting of FZD10, ITPR2, Rac1, ITPKC, DIAPH2, and others; and (2) Calcium ion homeostasis (5.446E-03) including RYR3, TRPM1, GNAI1, GNAS1 PTGER3, RXFP3, PDE2A, TRPM8, FZD10, TRPM1, IP3R2, GNAQ, P2RX3, ATP1, among others (see Supplementary Table 2). In the hippocampus of the P301S Tg mice, the functional networks showed: (1) Axon development (FDR = 1.596E-03) that was enriched by WNT11, ADCY3, ULK2, GAP43, TUBB, and others; as well as (2) Response to calcium ion (FDR = 2.249E-04) enriched by WNT11, ADCY3, RYR1, ACTG1, and others (for more information, see Supplementary Table 3).

We used STRING software to analyze protein-protein interactions among S-nitrosylated proteins in the P301S Tg mice. Physical interactomes related to synapse, calcium, and proteasome (Fig. 2e) were generated only in the Cor-Tg group with “high reliable interactions” score (>0.7), showing a correlation with the functional networks. The network analysis suggests that SNO-proteins are involved in synaptic and calcium processes, as well as ubiquitination (proteosomal) processes. Supplementary Figure 6 shows the interactome analysis of the four tested groups.

Finally, to test the association of the tau protein with the SNO-proteins, we built a network integrating tau using the Metacore software. We identified primary and secondary interactions of tau with the SNO-proteins in the Cor-Tg group (see Fig. 2f).

### Quantification of 3-nitrotyrosine (Ntyr) and nNOS in the hippocampal and cortical regions of WT and P301S Tg mice

As discussed elsewhere^39^, once NO is produced by the intracellular nitric oxide synthase, it undergoes a fast reaction with superoxide anion to form peroxynitrite (OONO−). Peroxynitrite is highly reactive and one of its major targets is tyrosine to which is modified into 3-nitrotyrosine (Ntyr)^39^. Therefore, Ntyr level is widely used as indicator of regional NO level. Here, we examined Ntyr levels in various cortical and hippocampal regions using IHC and morphometric quantification of Ntry intensity. This study focused on those brain regions that have functional relevance to the behavioral and physiological deficits in the P301S mouse model:^29,40^ CA1, CA2, CA3, and dentate gyrus regions of the hippocampus as well as the entorhinal cortex (EC). We also tested several other cortical regions known to be relevant in AD, such as the prefrontal cortex, motor cortex and somatosensory cortex^41,42^.

**Fig. 3:**
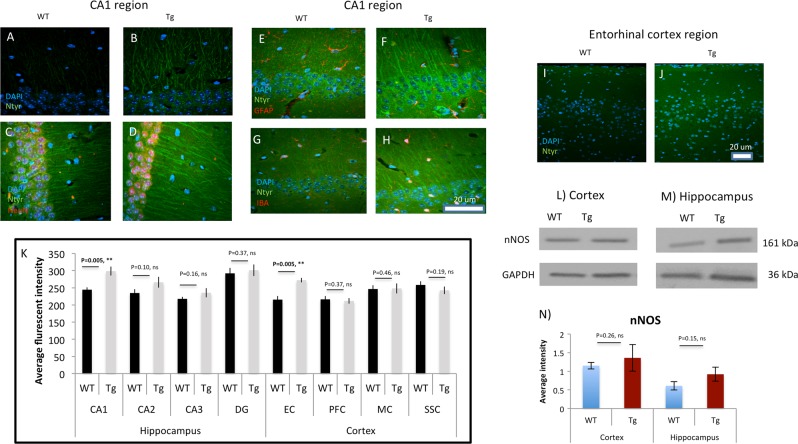
Significant increase of Ntyr levels in neruons in enthorhinal cortex (EC) and hippocampal CA1 region of P301S Tg mice compared to WT controls. **a**, **b** Representative immunohistochemistry staining of 3-nitrotyrosine (Ntyr) (green) and DAPI (blue) for CA1 regions. **c**, **d** Co-localizing Ntyr with neurons (NeuN-Red). **e**, **f** Co-localizing Ntyr with astrocytes (GFAP-Red). **g**, **h** Co-localizing Ntyr with microglia (IBA-Red). **i**–**j** Representative staining of Ntyr (green) and DAPI (blue) for entorhinal cortex (EC) region. **k** Quantitative analysis of the fluorescence intensity of Ntyr in the CA1, CA2, CA3, dentate gyrus (DC), EC, prefrontal cortex (PFC), motor cortex (MC), and somatosensory cortex (SSC) regions. Values presented are the mean and ±SEM. One tailed *t*-test was conducted. **p < 0.005, WT mice (*n* = 6) and Tg mice (*n* = 6). **l**, **m** Representative WB (for nNOS) from cortex and hippocampus tissues prepared from Cor-WT, Cor-Tg, Hip-WT, and Hip-Tg. **n** The relative average WB intensity of nNOS normalized to GAPDH. WT mice (*n* = 3) and Tg mice (*n* = 3)

We found a significant increase of Ntyr in the CA1 region (*p*-value < 0.005) in P301S Tg mice compared to WT controls (See Fig. 3a, b, k). A marginal increase was observed in CA2 (*p*-value = 0.10; Fig. 3k), whereas no difference was detected in the CA3 and DG regions (Fig. 3k). To determine which cell types are the major source of Ntyr in CA1, co-staining experiments were carried out using Ntyr and cell-type specific markers. We found that the vast majority of Ntyr in CA1 is within neurons (Fig. 3c, d) but not in astrocytes (Fig. 3e, f) or microglia (Fig. 3g, h). A similar expression pattern was observed in both WT and P301S Tg mice (Fig. 3c–h).

A significant increase in Ntyr levels was specifically observed in the EC (*p*-value < 0.005) in the P301S Tg mice relative to WT controls (See Fig. 3i–k)^43^.

We examined whether nNOS protein expression changed in the WT compared to Tg groups in both cortex and hippocampus tissues. A marginal increase (not significant) was found in Hip-Tg compared to Hip-WT (See Fig. 3m, n) and in Cor-Tg compared to WT-Tg (See Fig. 3l, n).

To test whether inducible NOS (iNOS) plays a role in the P301S mouse model, we measured its level of expression in the cortex and found no difference between WT and Tg mice (See Supplementary Figure 7).

### S-nitrosylation of RNF-213 alters non-canonical Ca++/Wnt signaling

Several proteins that regulate the NC-WCa, including FZD10, RNF-213, inositol-triphosphate 3-kinase C (ITPKC), inositol-triphosphate receptor 2 (ITPR2), rynaodine receptor 3 (RYR3), and guanine nucleotide-binding protein G (q) subunit alpha (GNAQ) were S-nitrosylated in the cortex of the P301S Tg mice.

**Fig. 4:**
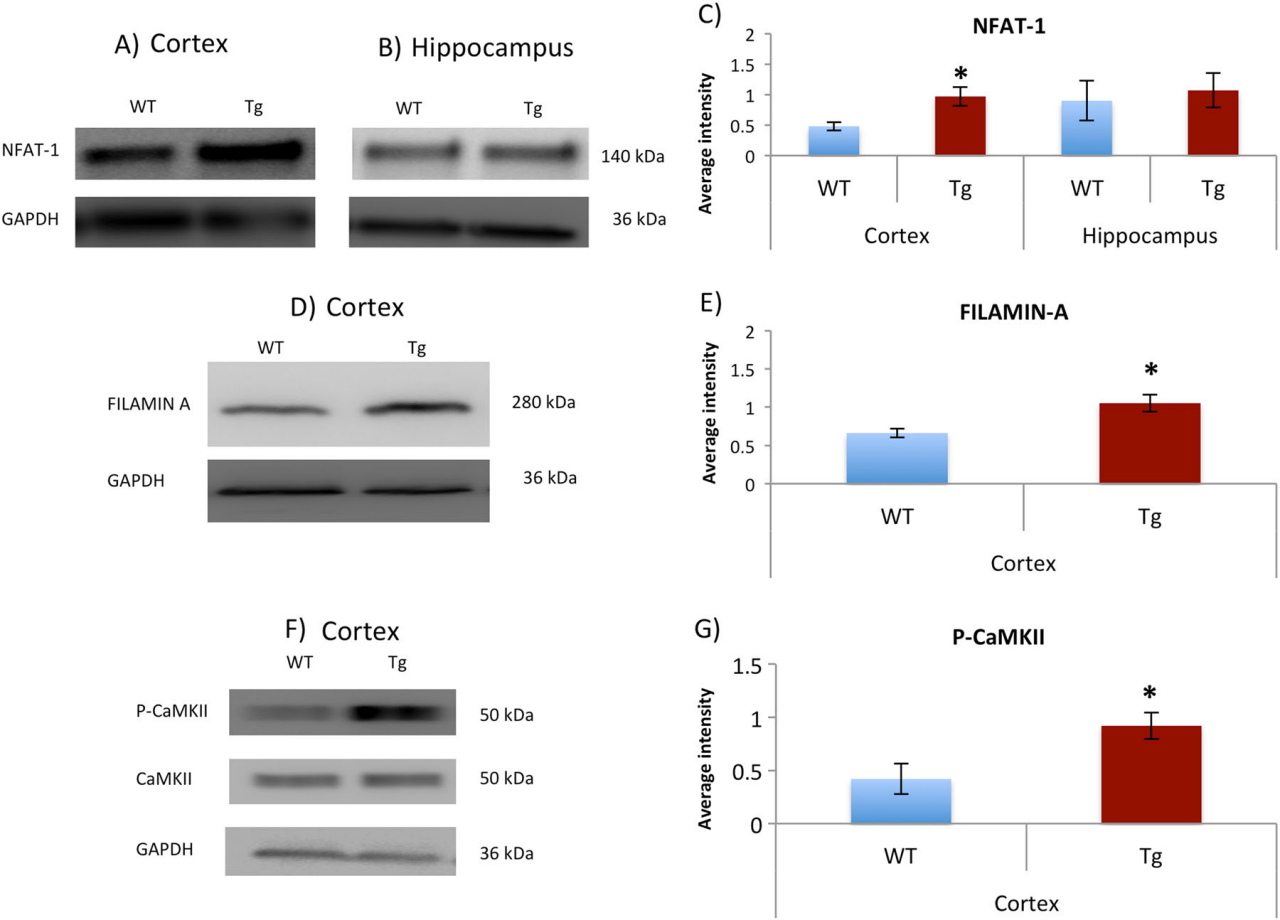
Significant increase of NFAT-1, FILAMIN-A, and P-CaMKII in cortex of P301S mice (Cor-Tg) compared to WT (Cor-WT). Immunoblotting analysis of NFAT-1 in (**a**) cortex and (**b**) hippocampus, (**d**) FILAMIN-A in cortex, and (**f**) P-CaMKII levels in the cortex of Tg mice and WT controls. Corresponding quantification of relative signal intensity for (**c**) NFAT-1, (**e**) FILAMIN A, and (**g**) P-CaMKII normalized to GAPDH, and CaMKII in (**h**). The data are presented as mean values ± SEM. One tailed t-test was conducted. *p < 0.05. WT mice (*n* = 3) and Tg mice (*n* = 3); each n is a pooling of three mice

RNF-213 is an E3 ubiquitin-protein ligase that targets NFAT-1, a downstream transcription factor, and FILAMIN-A, for proteasomal degradation attenuating the NC-WCa^44^. Several studies have previously reported that S-nitrosylation of different ligases, such as RNF-25 and PARKIN, leads to its auto-ubiquitantion and, as a consequence, increases its substrates levels^45,46^. We tested two different RNF-213 substrates, NFAT-1 and FILAMIN-A, and found a significant increase in the levels of both substrates in the cortex of the P301S mice (See Fig. 4a, c–e). No differences in NFAT-1 levels were found in the hippocampus region, since SNO-RNF213 was found in the hippocampus of both the P301S Tg mice as well as WT controls (Fig. 4b, c). FILAMIN-A bands were not detected in the hippocampus of both groups.

We examined other components of the pathway for possible alterations. It has previously been reported that calcium leads to activation of the NC-WCa via increased auto-phosphorylation of CaMKII^33^. We found SNO-ITPKC, SNO-ITPR2, and SNO-RYR3 in Cor-Tg, which may lead to further calcium release and leak from the ER (See Discussion). Therefore we measured phosphorylated CaMKII on threonine 286 as an indication of calcium increase and activation of the NC-WCa pathway. We found a significant increase in P-CaMKII in Cor-Tg (see Fig. 4f, g).

To validate that this enrichment is specific to the NC-WCa pathway and not to other non-canonical Wnt pathways, we measured P-CREB, which is not a unique protein for specific Wnt pathway. Supplementary Figure 8 shows that there is no difference between the Tg and WT groups both in P-CREB and CREB, which supports our conclusions.

Furthermore, Supplementary Figure 9 presents the KEGG analysis of three different Wnt signaling pathways and shows the specificity of CaMKll as an upstream protein that activates the NC-WCa pathway. It also shows NFAT as a downstream protein in this pathway and both were increased in our data.

## Discussion

Our study combines state-of-the-art proteomic as well as biological methods to study changes in the S-nitroso-proteome and the consequences on biological processes and signaling pathways in the P301S Tg mouse model of tauopathy. We examined the hippocampus and the cortex of two-month-old mice, which is the earliest age tested in this model (see Fig. 1).

GO and pathway analysis of the S-nitrosoproteome in the P301S Tg mice revealed enrichment of processes and pathways that are known to be affected in AD, pointing to the possibility that S-nitrosylation plays an important role in the neuropathology. For example, in both cortex and hippocampus of the P301S mice, we found enrichment of SNO-proteins associated with calcium-related processes (see Fig. 2), indicative of an association between S-nitrosylation, calcium, and neurodegeneration. This correlates with previous findings which support the theory that Ca++ dysregulation plays a major role in AD^47,48^. Other neuronal, synaptic, and vesicle processes were enriched in either or both regions such regulation of neurotransmitter, neurogenesis, nervous system development, and vesicle mediated transport, which is consistent with results from previous studies showing their involvement in AD^49,50^.

In the CK-p25–inducible mouse model of AD, proteins related to amyloid precursor protein processing and secretion are S-nitrosylated, correlating with increased amyloid formation and tau aggregates^17^. We compared the results from the CK-p25 to the P310S model to examine whether there is a convergence of processes and pathways (GO and KEGG). Neuronal and calcium related processes were enriched in the cortex of both models, including calcium signaling pathways, neuronal differentiation, neuron projection, and vesicle mediated transport (see Fig. 2). Endosomal pathway abnormalities were previously reported in AD^51,52^, which is correlated with our findings in both models. This suggests that SNO may be central to AD pathology, especially in calcium deregulation^47,48^. Since both models showed similarity in behavioral deficits of memory and spatial learning^53,54^, different genetic defects in AD may lead to common enzymatic deficits that converge into altered biochemical pathways.

**Fig. 5:**
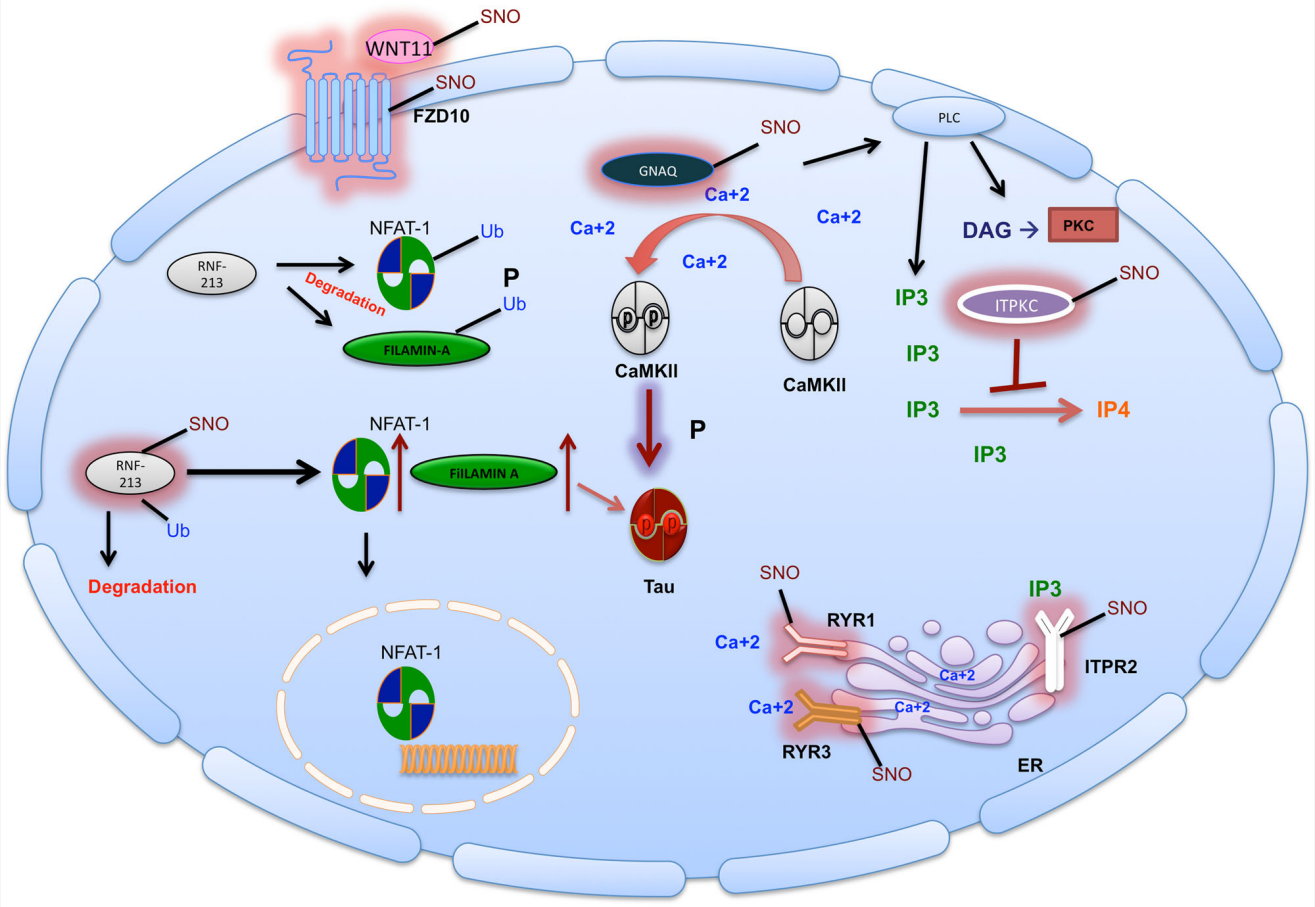
S-nitrosylation alters the non-canonical Wnt/calcium signaling pathway. Right side: SNO of RYR lead to Ca++ release from the ER. SNO of IP3R may also lead to Ca++ out-flux from the ER. ITPKC, the enzyme that converts IP3 to IP4, when SNOed leads to the its inhibition. SNO-ITPKC leads to increase of IP3 that activates IP3R. Altogether, this leads to increase of Ca++ release from the ER. Middle: Ca++ release from the ER as a consequence of SNO of different proteins as indicated above, activates CaMKII and leads to increase of its auto-phosphorylation and activation. As occurs in tauopathy, CaMKII hyper-phosphorylates tau. Left: RNF-213 is an E3 ubiquitin-ligase and targets NFAT-1 and FILAMIN-A for proteosomal degradation and attenuates non-canonical WNT/Ca+2 signaling. However, SNO-RNF213 leads to its auto-ubiquitination and degradation. This leads to an increase of NFAT-1 and FILAMIN-A levels (RNF-213 substrates). Note: Several proteins in this pathway were SNOed: WNT11 is mainly an activator of the non-canonical pathway; FZD10; GNAQ that activates PLC

Specifically, we found the non-canonical Wnt signaling process was enriched in the GO analysis of the Cor-Tg. Furthermore, other processes related to the non-canonical Wnt pathway were also enriched, such as signal transduction_IP3 signaling (see Supplementary Figure 3) and Ca++ dependent NFAT signaling. The NC-WCa pathway regulates intrinsic properties of neurons, mobilizes intracellular calcium and regulates key components in synapses^55^. Since we found key proteins in the pathway were S-nitrosylated, which may alter the pathway’s normal function, we consider their involvement in the NC-WCa in the P301S Tg mice. ITPKC which was S-nitrosylated in the Cor-Tg mice plays an important role in the NC-WCa. ITPKC phosphorylates inositol 2,4,5-triphosphate (IP3) to inositol 2,4,5,6-tetraphosphate (IP4). IP3 acts on IP3R leading to calcium release and activation of the NC-WCa^55^. Most studies have reported that the effect of S-nitrosylation on kinase activity is inhibitory and the inhibition may be exerted directly through suppression of kinase activity or by modulating the interaction of kinase and substrate (reviewed in^56^). We therefore suggest that S-nitrosylation of ITPKC inhibits its activity and leads to increase in IP3 levels which leads to Ca++ release from the ER (see Fig. 5). Another calcium-related component that was S-nitrosylated in the Cor-Tg is RYR3. A previous investigation has shown that SNO of RYR leads to calcium leak from the ER^57^, which indicates the possibility that SNO-Ryr3 may also lead to Ca++ leak. Ca++ release in turn has been shown to lead to increase of CaMKII autophosphorylation^35^, which facilitates activation of the NC-WCa signaling pathway^34^. Indeed, we observed increased levels of phosphorylated CaMKII consistent with Ca++ dependent activation of the NC-WCa signaling in the P301S Tg mouse. Active CaMKII (P-CaMKII) induces activation of the NFAT-1 which regulates cell adhesion and migration^34^, neuronal growth and axon guidance in both the developing and adult nervous system^58^. Importantly, CaMKII activation also leads to phosphorylation of the tau protein on Thr212, Ser214, Ser262, and Ser356 residues^59^. Another study showed that CaMKII dysregulation in Alzheimer’s patients may be a modulator of toxicity in Alzheimer’s disease, a dementia characterized by aberrant calcium signaling, synapse and neuronal loss, and impaired memory^60^. A Different study showed that disruption of calcium homeostasis and the downstream kinase CaMKII coincides with pathological phosphorylation of tau in AD brains^61^.

As previously reported, RNF-213 is inhibited by auto-ubiquitination upon S-nitrosylation^45,46^, and thus leads to activation of NC-WCa pathway, by reduced turnover of its substrates, NFAT-1 and FILAMIN-A. Indeed, the levels of both NFAT-1 and FILAMIN-A, were increased in the cortex of the P301S mice, consistent with activation of NC-WCa signaling pathway. Our results correlate with previous findings showing accumulation and increased levels of NFAT isoforms in post-mortem brains from patients with AD, which further suggest that changes in NFAT levels contribute to AD progression;^62^ The fact that they found increase of NFAT in both early and late stage of the disease suggests that our early molecular findings are linked to the later disease mechanism as well. Other recent studies also showed an increase of NFAT-1 in a different transgenic AD mouse model^63^, and a third proteopathy for AD, suggests that an alteration of the scaffolding protein FILAMIN-A, is critically linked to the amyloid and tau pathologies in AD and leads to increased tau phosphorylation^64^. Another study showed that FILAMIN-A inhibitors decrease P-Tau and amyloid-beta aggregates^65^. These studies confirm the involvement of NFAT-1 and FILAMIN-A in AD pathology.

Our study demonstrates that S-nitrosylation of key proteins activates the NC-WCa signaling pathway as measured by increased phosphorylation of CaMKII in the cortex of the P301S mouse model, and this is likely through increased calcium release from the ER. Moreover, S-nitrosylation of RNF-213 led to an increase in NFAT-1 and FILAMIN-A levels in the cortex of P301S mice, further amplifying the NC-WCa signaling pathway (See Fig. 5). Taken together, these findings provide powerful evidence implicating S-nitrosylation mediated alterations of the NC-WCa pathway in tauopathy.

Consistent with the changes in S-nitrosylation we observed a significant increase of Ntyr in CA1 and EC, which indicates an elevation of reactive oxygen species and nitric oxide formation, and has been widely reported in the AD literature (see reviews^66,67^). It is also well known that the EC region projects to the CA regions^43^. Moreover CA1 and EC have previously been shown to be a focal point for tau pathology and also the most heavily damaged regions in AD^29^. Therefore the significant increase of Ntyr in CA1 and EC regions, further validates the specificity of these regions in tauopathay.

We show in Fig. 3 that there may be a marginal, but not statistically significant, increase of nNOS levels in Tg compared to WT in both cortex and hippocampus. Further, an increase of free calcium (validated by increase of P-CaMKll) binding to calmodulin could lead to an increase in NO formation from nNOS. In addition, nNOS is found exclusively in neurons^68^ and proximity is important, because of the high diffusivity and reactivity of NO. To test whether iNOS may play a role in the P301S mouse model, we measured its level of expression in the cortex and found no difference between WT and Tg mice. We favor nNOS as the source of S-nitrosylation in these experiments because it is localized in neurons, but there is no basis for a definitive answer.

In conclusion, we have identified dysregulation of the NC-WCa signaling pathway as an early molecular sign of tau pathology in the cortex of the P301S mouse model. Specifically, we demonstrated that NC-WCa is aberrantly activated in early stages of disease progression. Our study provides a novel insight into the contribution of S-nitrosylation to tau pathology and suggests it mediates the dysregulation of NC-WCa. The increase in P-CaMKII, NFAT-1, and FILAMIN-A are correlated with human data discussed above which will allow future translational studies of potential drug targets for treatment of neurodegenerative diseases.

## Supplementary information

Supp. information

Supplementary Figure 1

Supplementary Figure 2

Supplementary Figure 3

Supplementary Figure 4

Supplementary Figure 5

Supplementary Figure 6

Supplementary Figure 7

Supplementary Figure 8

Supplementary Figure 9

Supp. table 1

Supp. table 2

Supp. table 3
