# Supplementary material for: S-nitrosylation of E3 ubiquitin-protein ligase RNF213 alters non-canonical Wnt/Ca+2 signaling in the P301S mouse model of tauopathy
Source: Transl Psychiatry. 2019 Jan 29;9:44. doi: 10.1038/s41398-019-0388-7 (PMC6351542; doi:10.1038/s41398-019-0388-7)
Supplement: Supplementary file 1 — Supp. information [file 41398_2019_388_MOESM1_ESM.docx]

**Supplementary Information**

***S-nitrosylation of E3 ubiquitin-protein ligase RNF213 alters non-canonical Wnt/Ca+2 signaling in the P301S mouse model of tauopathy***

Haitham Amal^a^, Guanyu Gong^a^, Elizabeta Gjoneska^b^, John S. Wishnok^a^, Li-Huei Tsai^b^, Steven R. Tannenbaum^a,c^

^a^ Department of Biological Engineering, Massachusetts Institute of Technology, Cambridge, MA 02139;

^b^ The Picower Institute for Learning and Memory, Department of Brain and Cognitive Sciences, Massachusetts Institute of Technology, Cambridge, MA 02139. ^c^ Department of Chemistry, Massachusetts Institute of Technology, Cambridge, MA 02139, USA.

Supplementary Figures:

Supplementary Figure 1: Plots of the number of proteins identified for each subset of 4 replicates: A) Cortex-WT, B) Cortex-Tg, C) Hipp-WT, D) Hipp-Tg


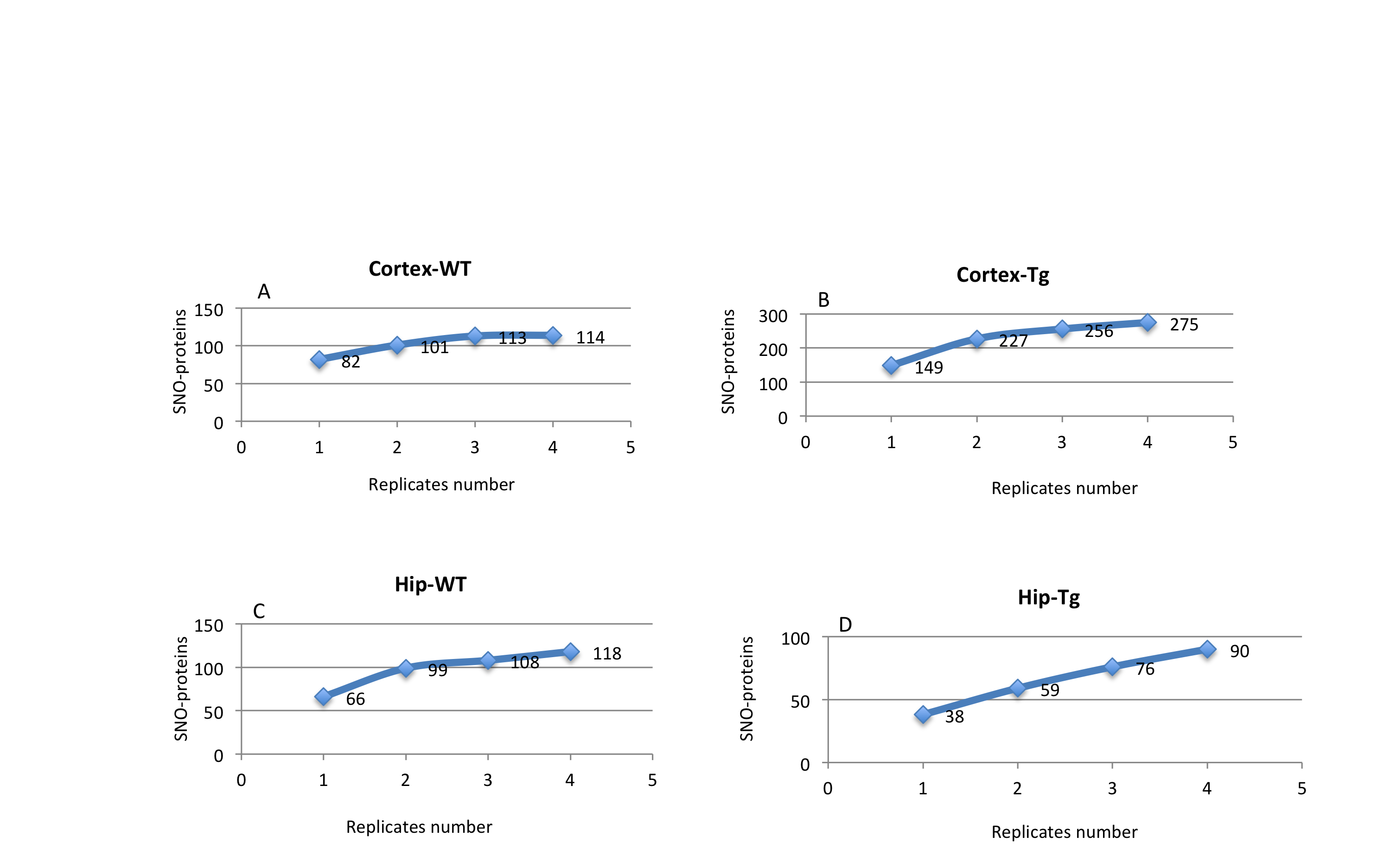


Supplementary Figure 2: Analysis by protein function using Metcaore software: A) Cortex-WT, B) Cortex-Tg, C) Hip-WT, D) Hipp-Tg


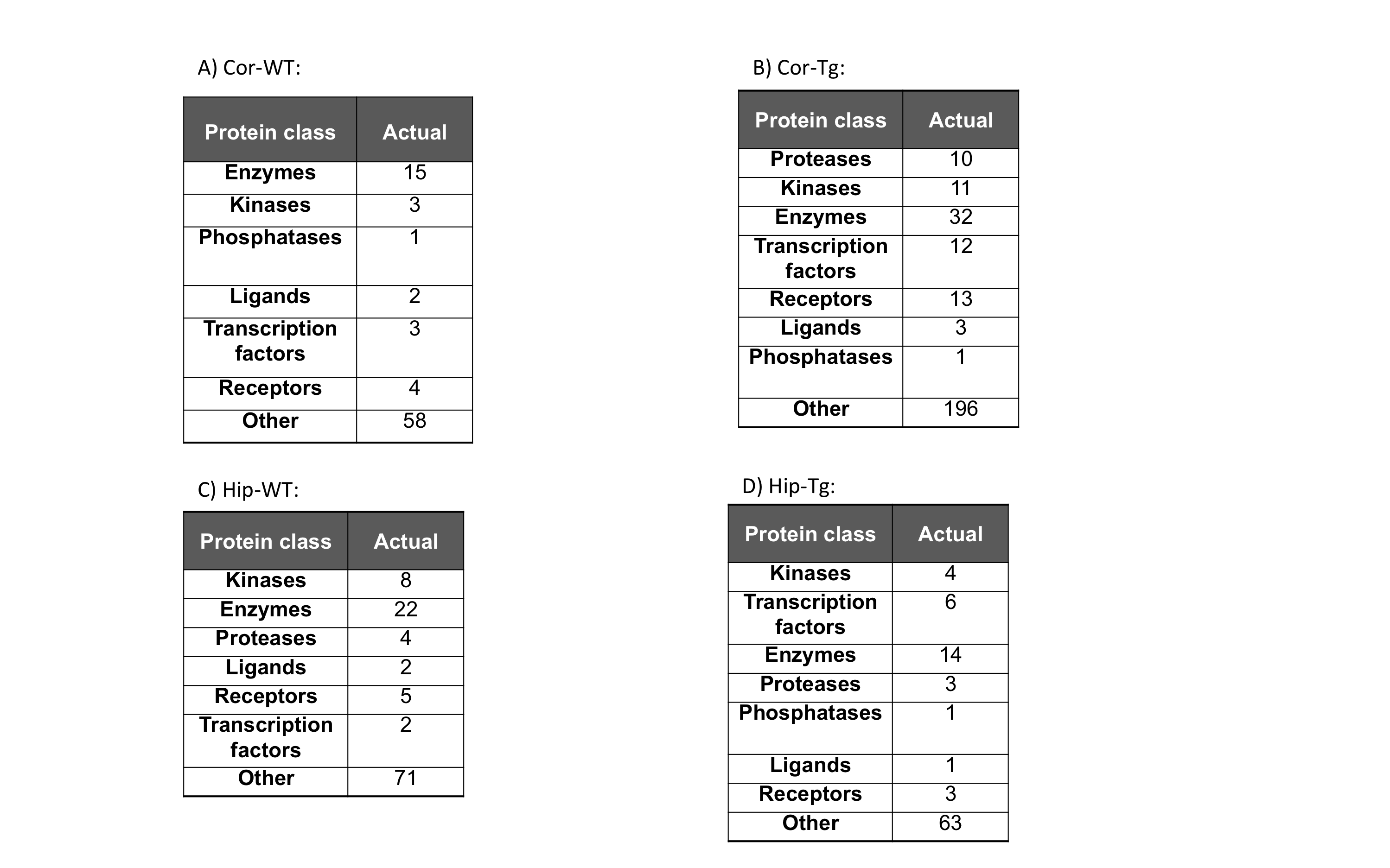


Supplementary Figure 3: Enriched IP-3 signaling pathway in Cor-Tg, red circled are S-nitrosylated proteins.


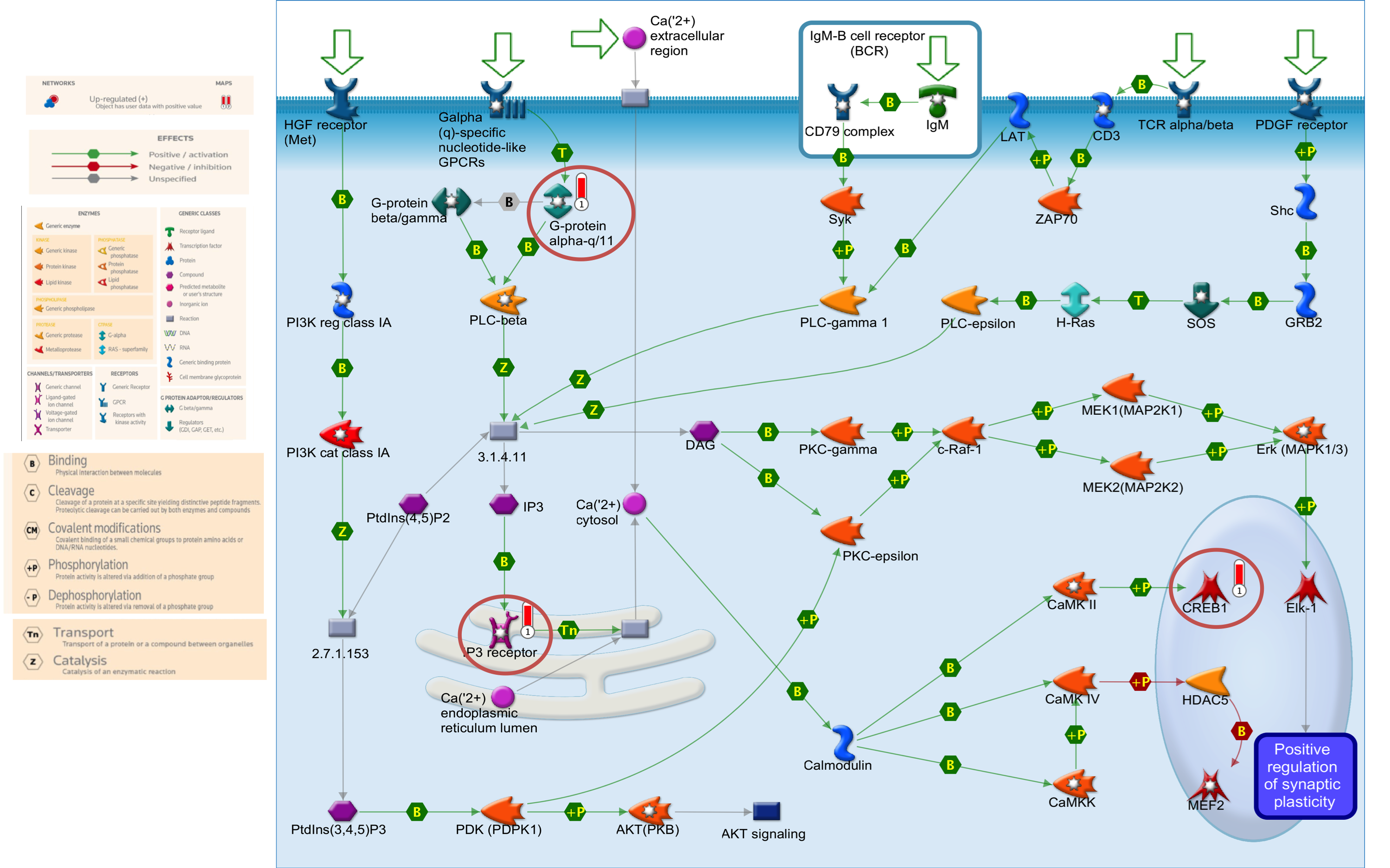


Supplementary Figure 4: Enriched Regulated NMDAR trafficking pathway in Cor-Tg, red circled are S-nitrosylated proteins.


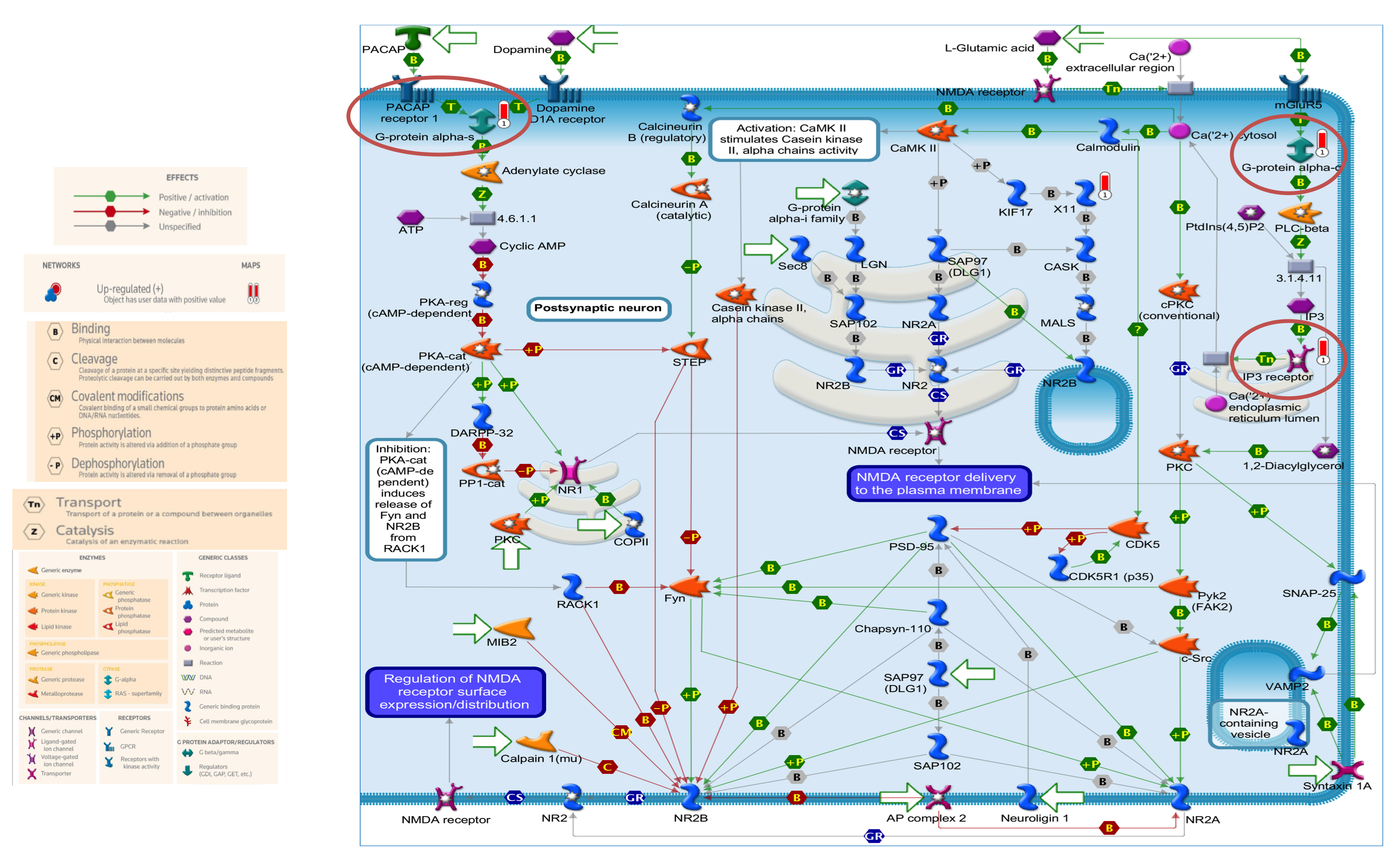


Supplementary Figure 5: Enriched Axon Growth pathway in Hip-Tg, red circled are S-nitrosylated proteins.


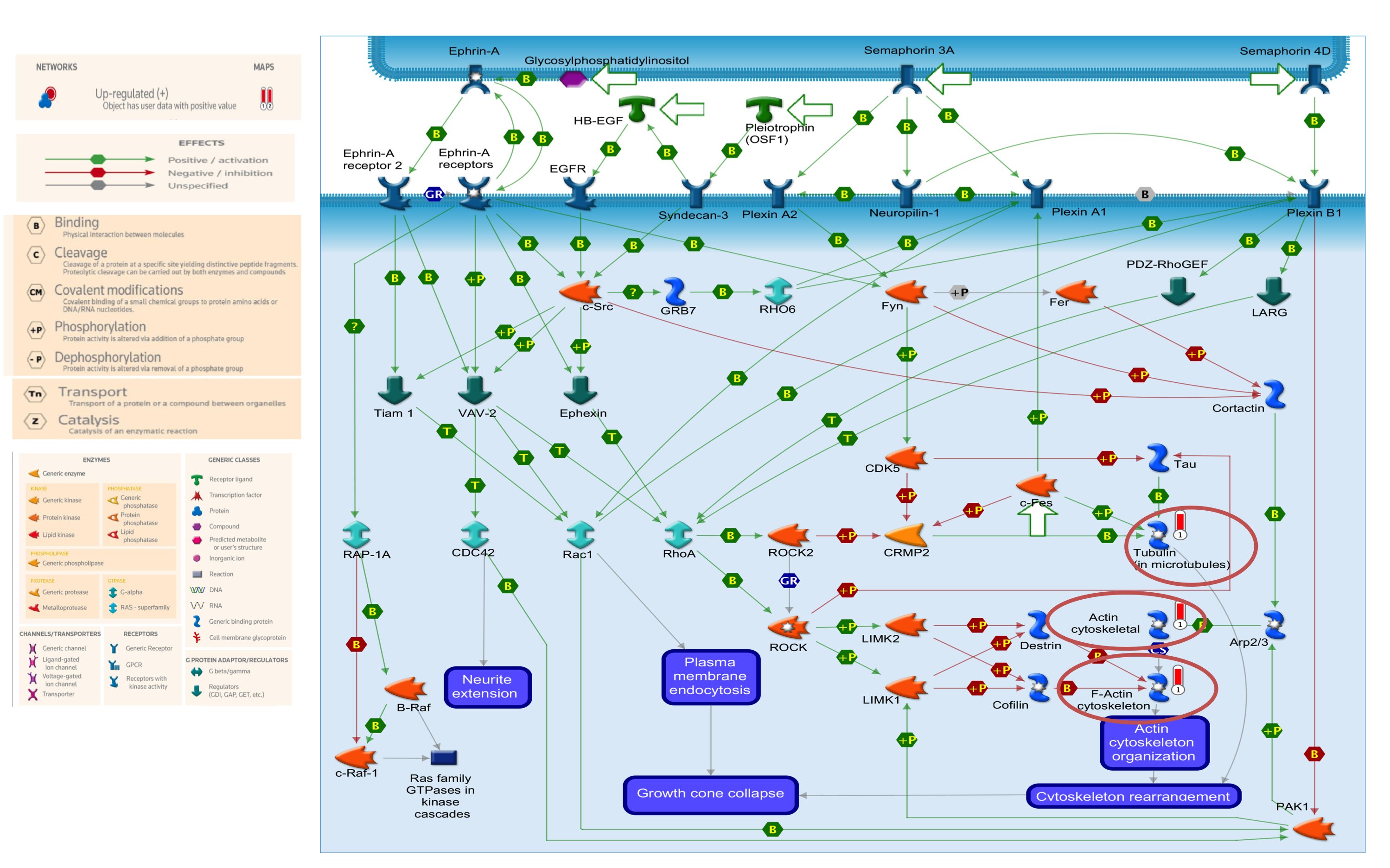


Supplementary Figure 6: Interactome analysis of the four tested groups.


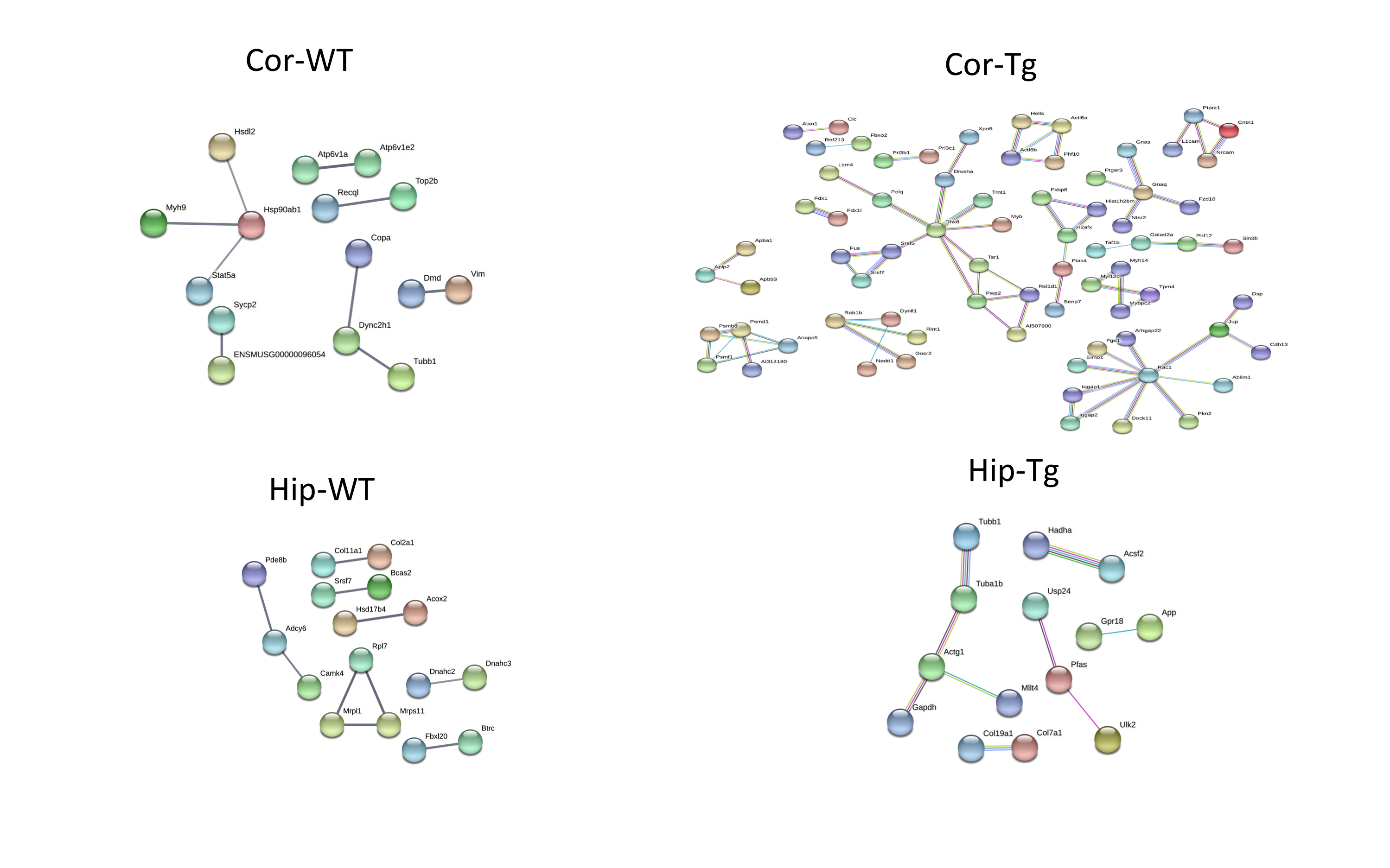


Supplementary Figure 7: Representative WB (for iNOS) from cortex tissues prepared from Cor-WT and Cor-Tg. The relative average WB intensity of iNOS comparing Cor-WT to Cor-Tg. The data is normalized to GAPDH and presented as mean +/- SEM. One tailed t-test was conducted.


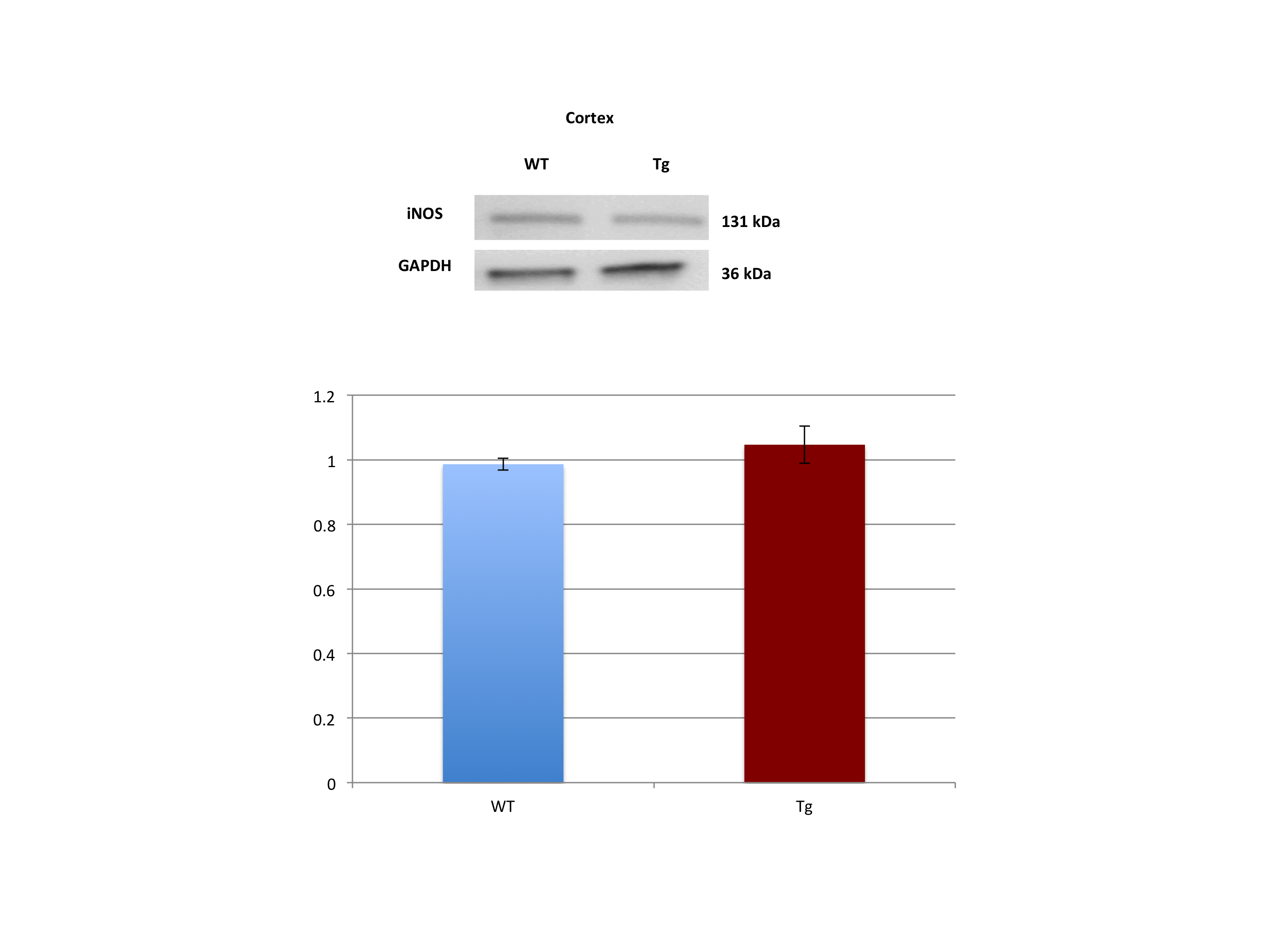


Supplementary Figure 8: The relative average WB intensity of P-CREB comparing Cor-WT to Cor-Tg and Hip-WT to Hip-Tg. The data is normalized to CREB and GAPDH and presented as mean +/- SEM. One tailed t-test was conducted. WT mice (n=3) and Tg mice (n=3), each n is pooling of 3 mice.


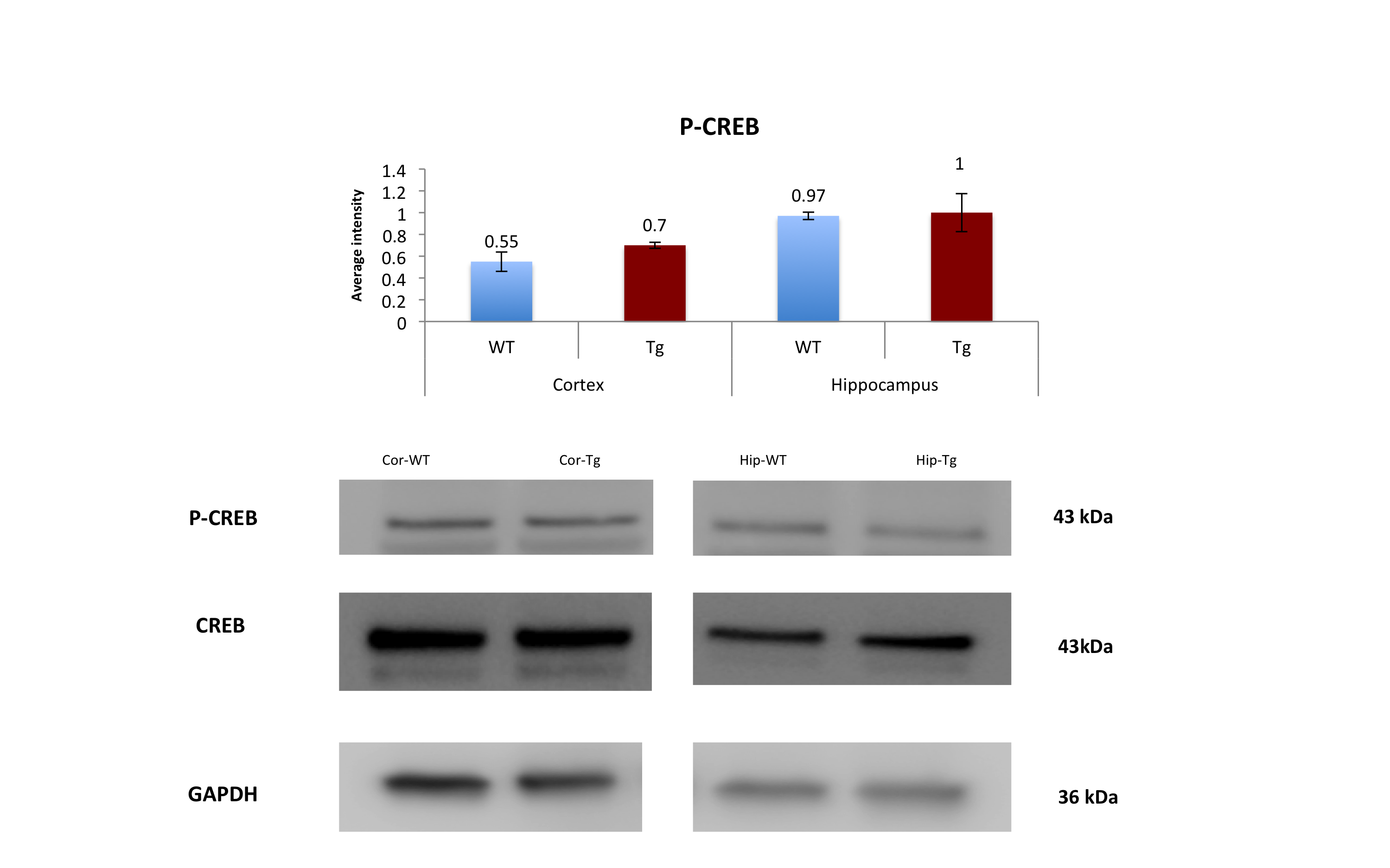


Supplementary Figure 9: KEGG analysis of the Wnt signaling pathway.


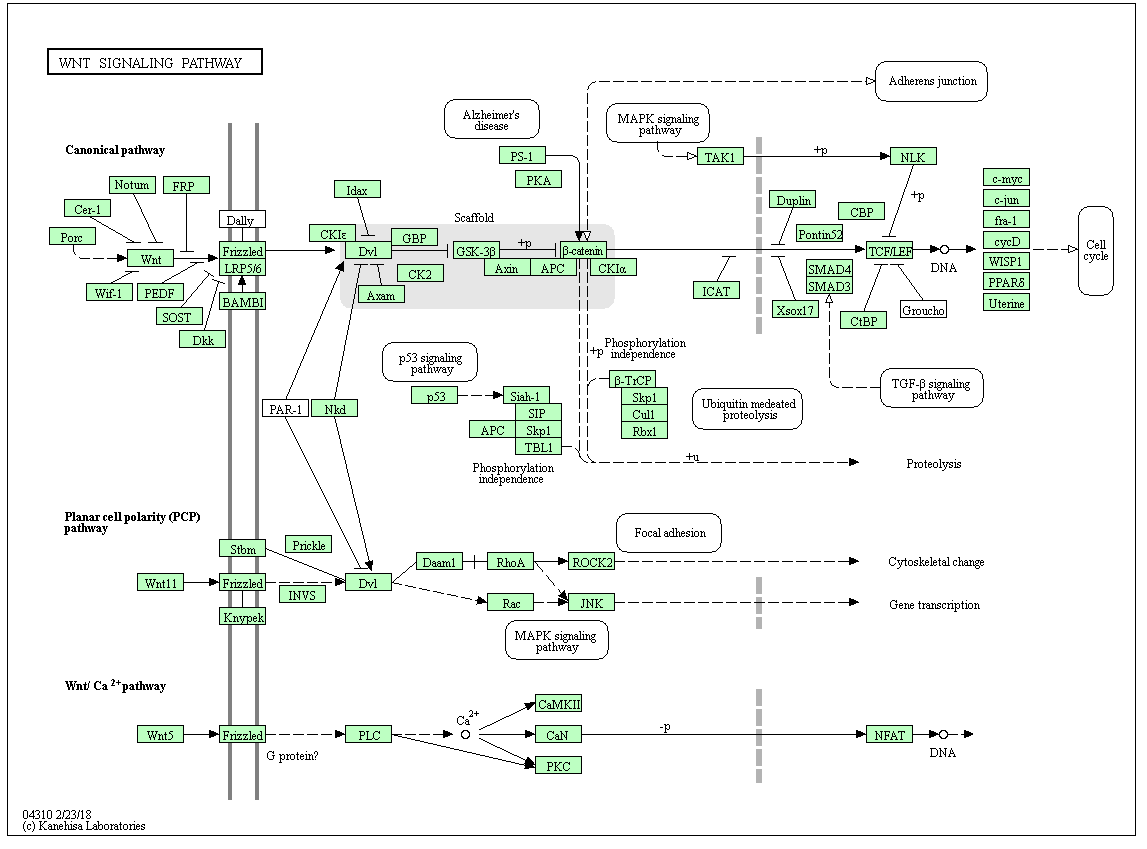


Supplementary Tables are uploaded as Excel files:

Table 1: IDs of SNO-protein in the four different groups.

Table 2: GO and pathways analysis of the Cor-WT and Cor-Tg.

Table 3: GO and pathways analysis of the Hip-WT and Hip-Tg.
