# Supplementary figures and images for: S-nitrosylation of E3 ubiquitin-protein ligase RNF213 alters non-canonical Wnt/Ca+2 signaling in the P301S mouse model of tauopathy
Source: Transl Psychiatry. 2019 Jan 29;9:44. doi: 10.1038/s41398-019-0388-7 (PMC6351542; doi:10.1038/s41398-019-0388-7)

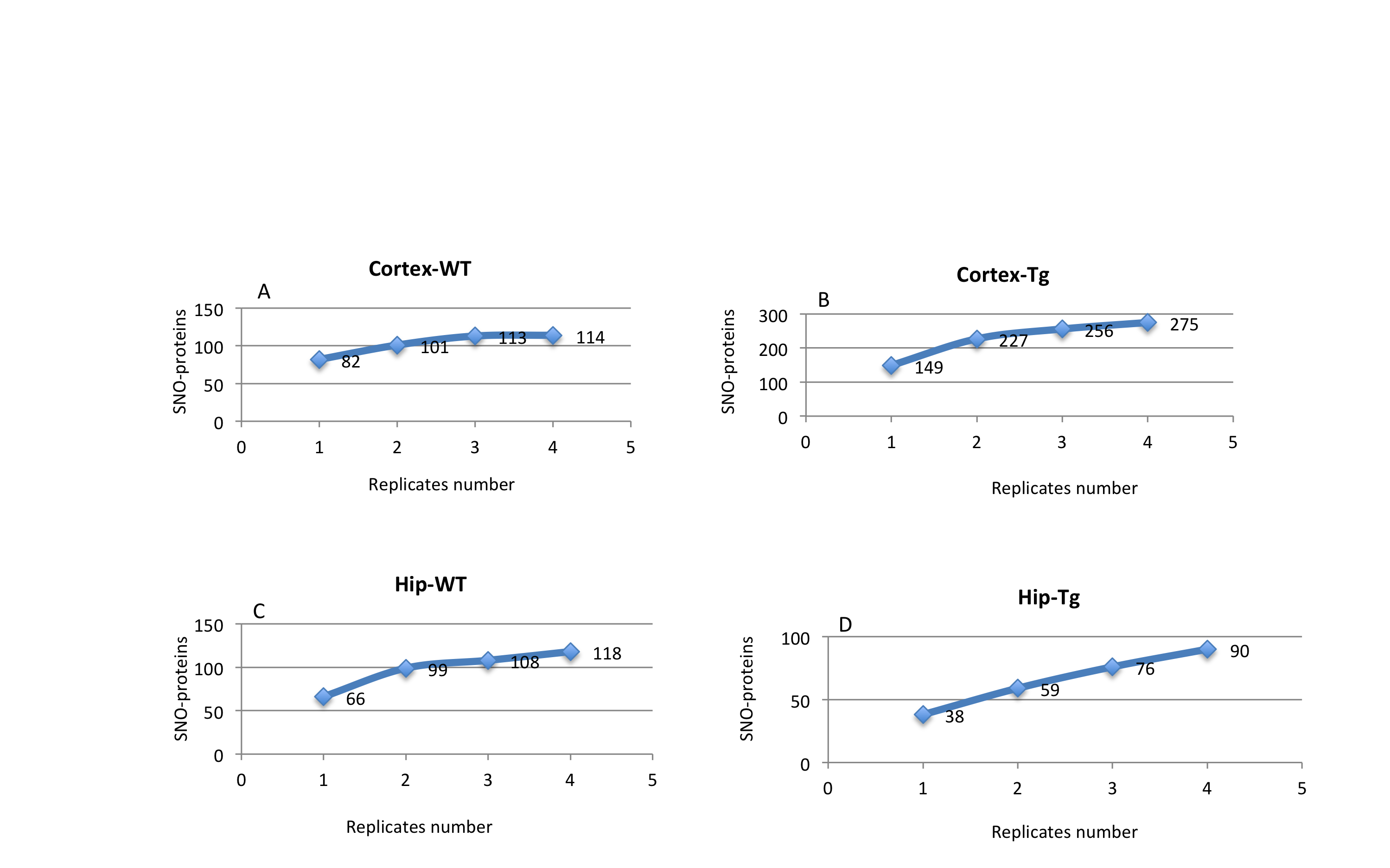

Supplement: Supplementary file 2 — Supplementary Figure 1 [file 41398_2019_388_MOESM2_ESM.tif]

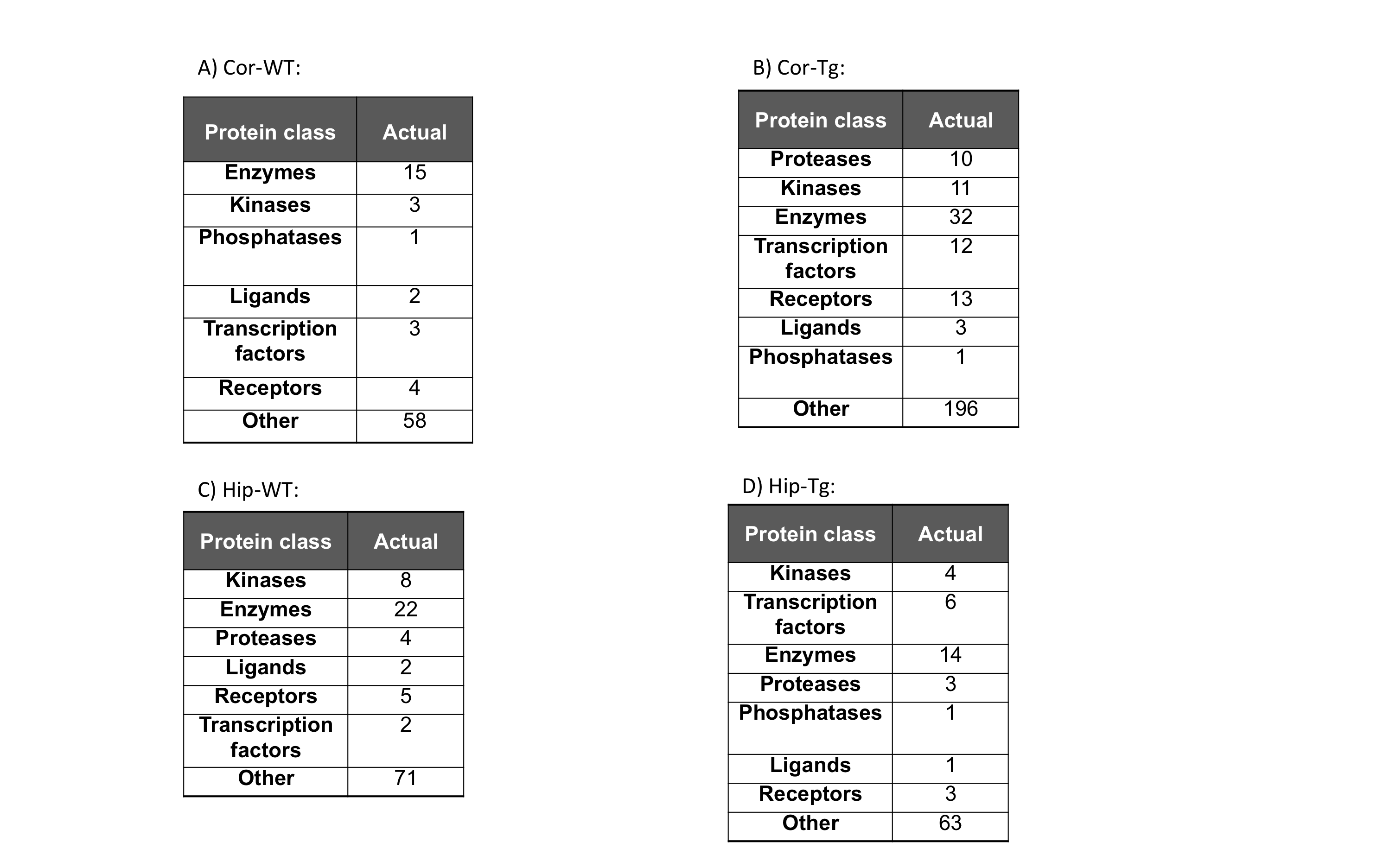

Supplement: Supplementary file 3 — Supplementary Figure 2 [file 41398_2019_388_MOESM3_ESM.tif]

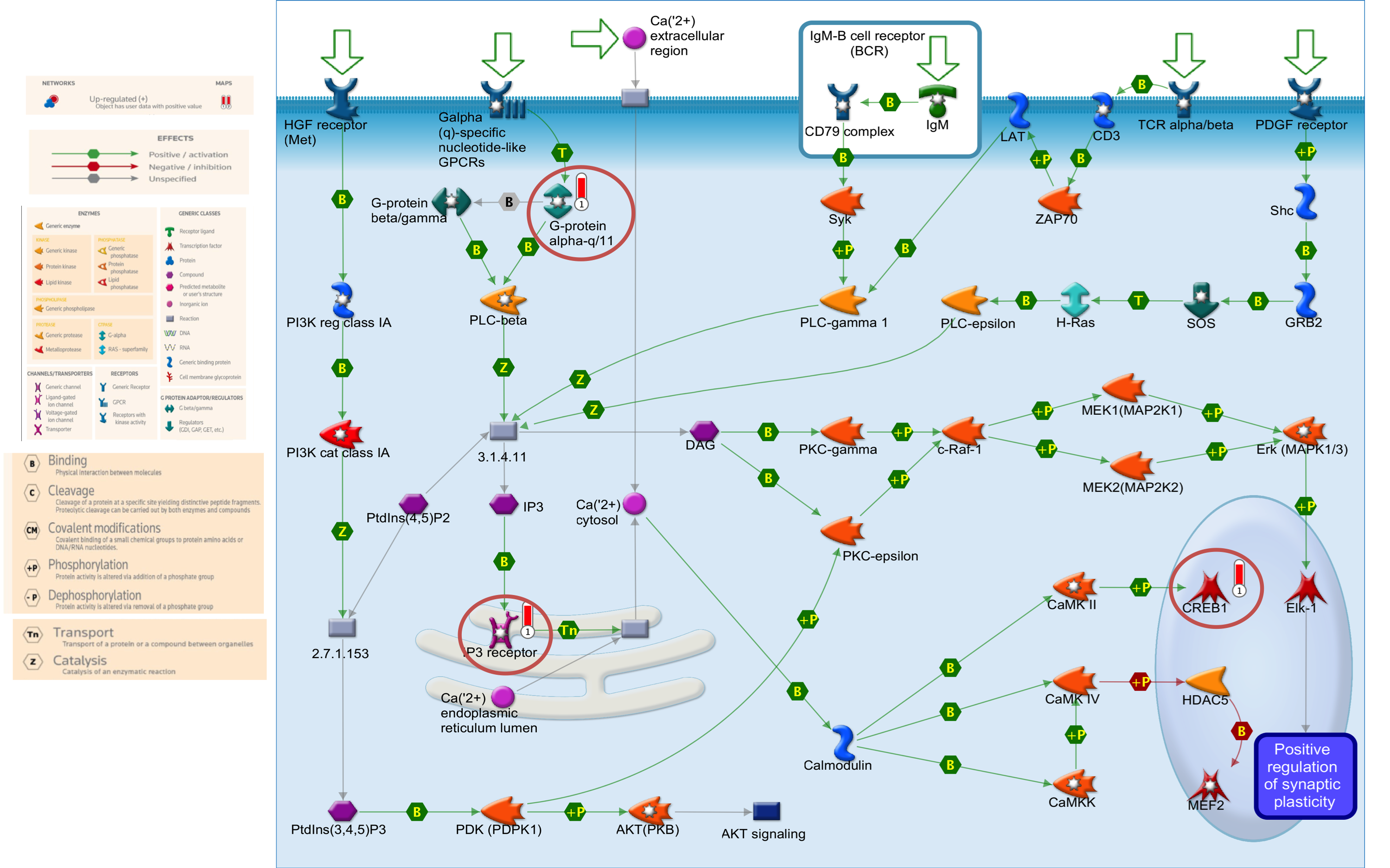

Supplement: Supplementary file 4 — Supplementary Figure 3 [file 41398_2019_388_MOESM4_ESM.tif]

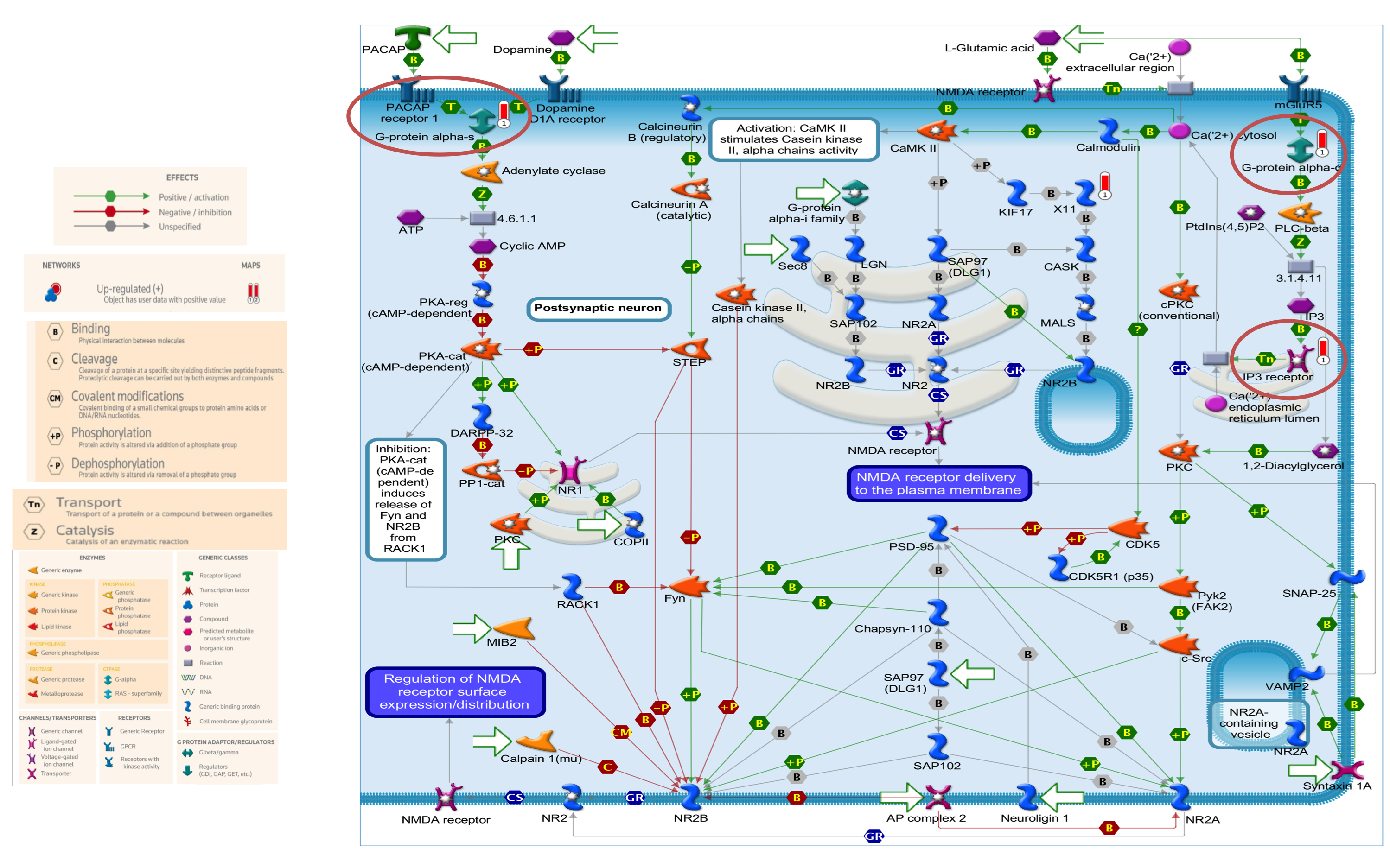

Supplement: Supplementary file 5 — Supplementary Figure 4 [file 41398_2019_388_MOESM5_ESM.tif]

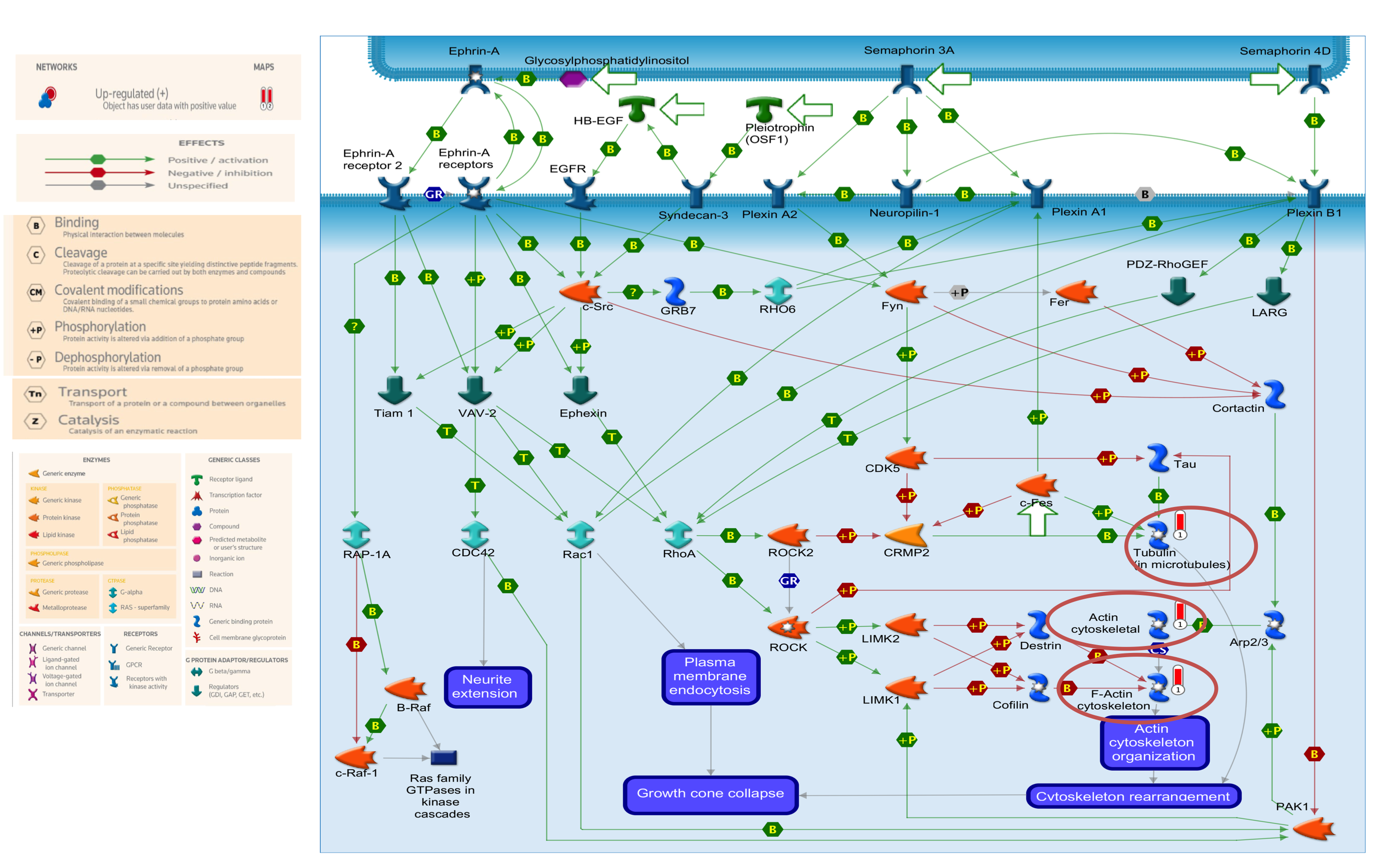

Supplement: Supplementary file 6 — Supplementary Figure 5 [file 41398_2019_388_MOESM6_ESM.tif]

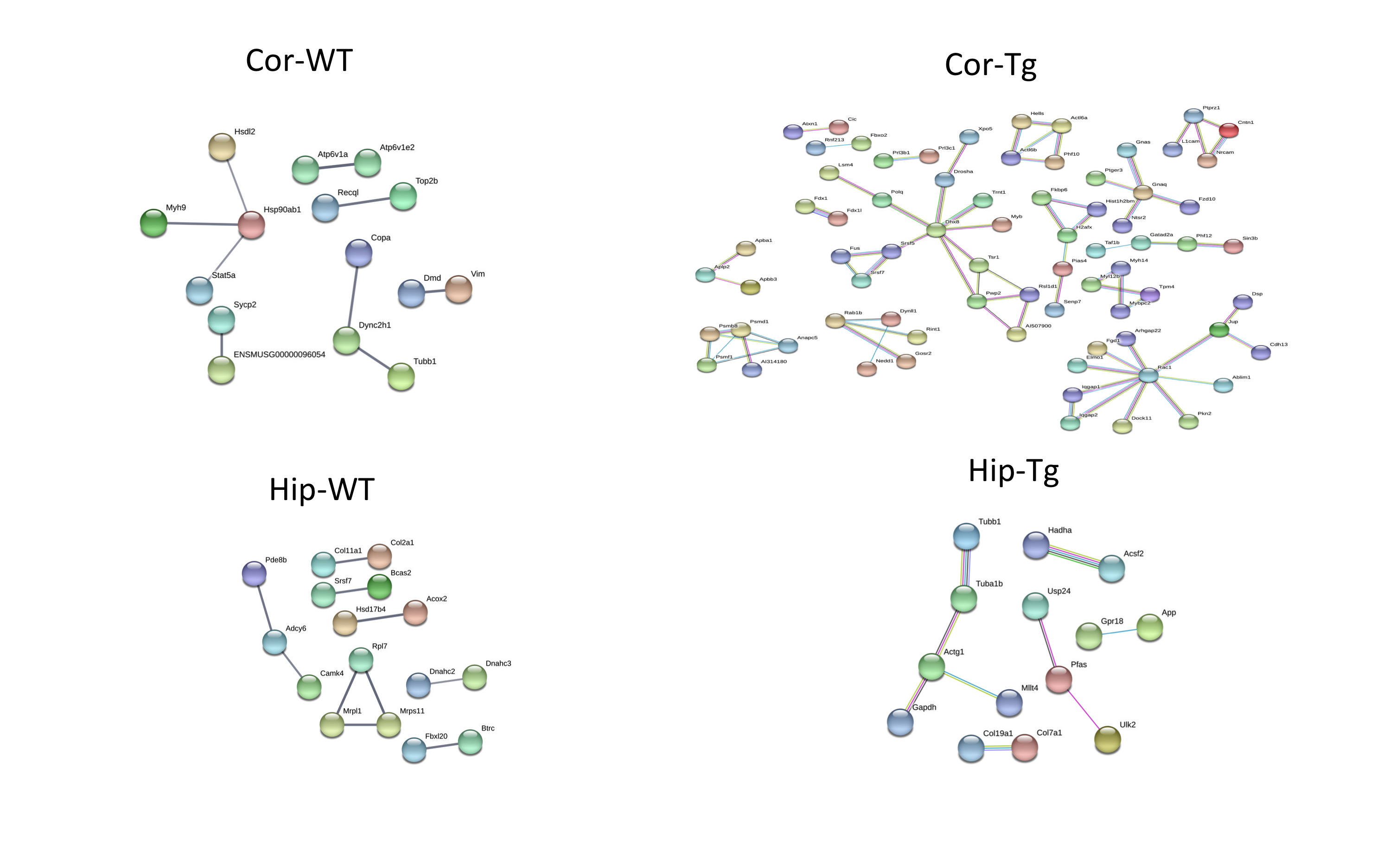

Supplement: Supplementary file 7 — Supplementary Figure 6 [file 41398_2019_388_MOESM7_ESM.tif]

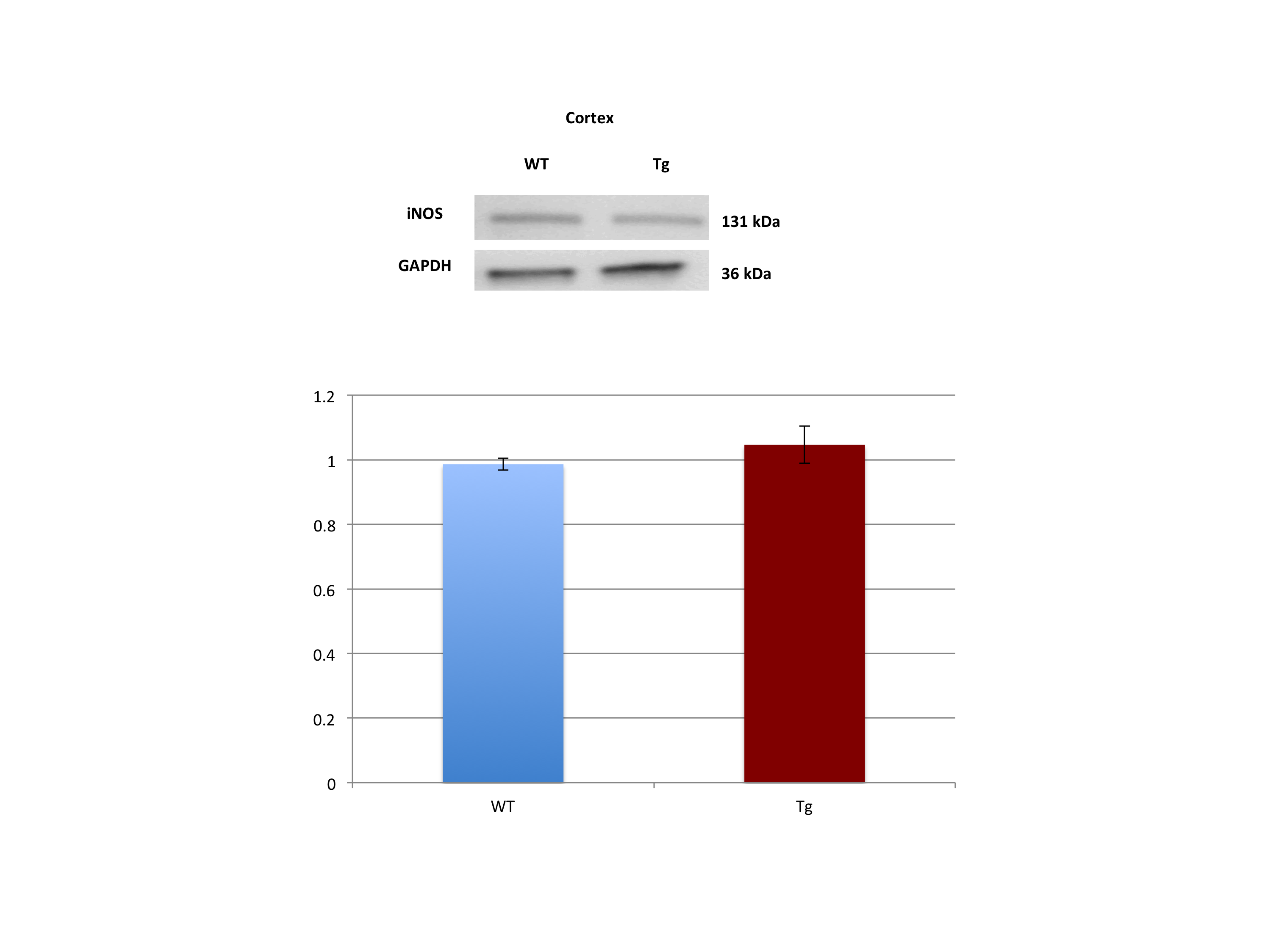

Supplement: Supplementary file 8 — Supplementary Figure 7 [file 41398_2019_388_MOESM8_ESM.tif]

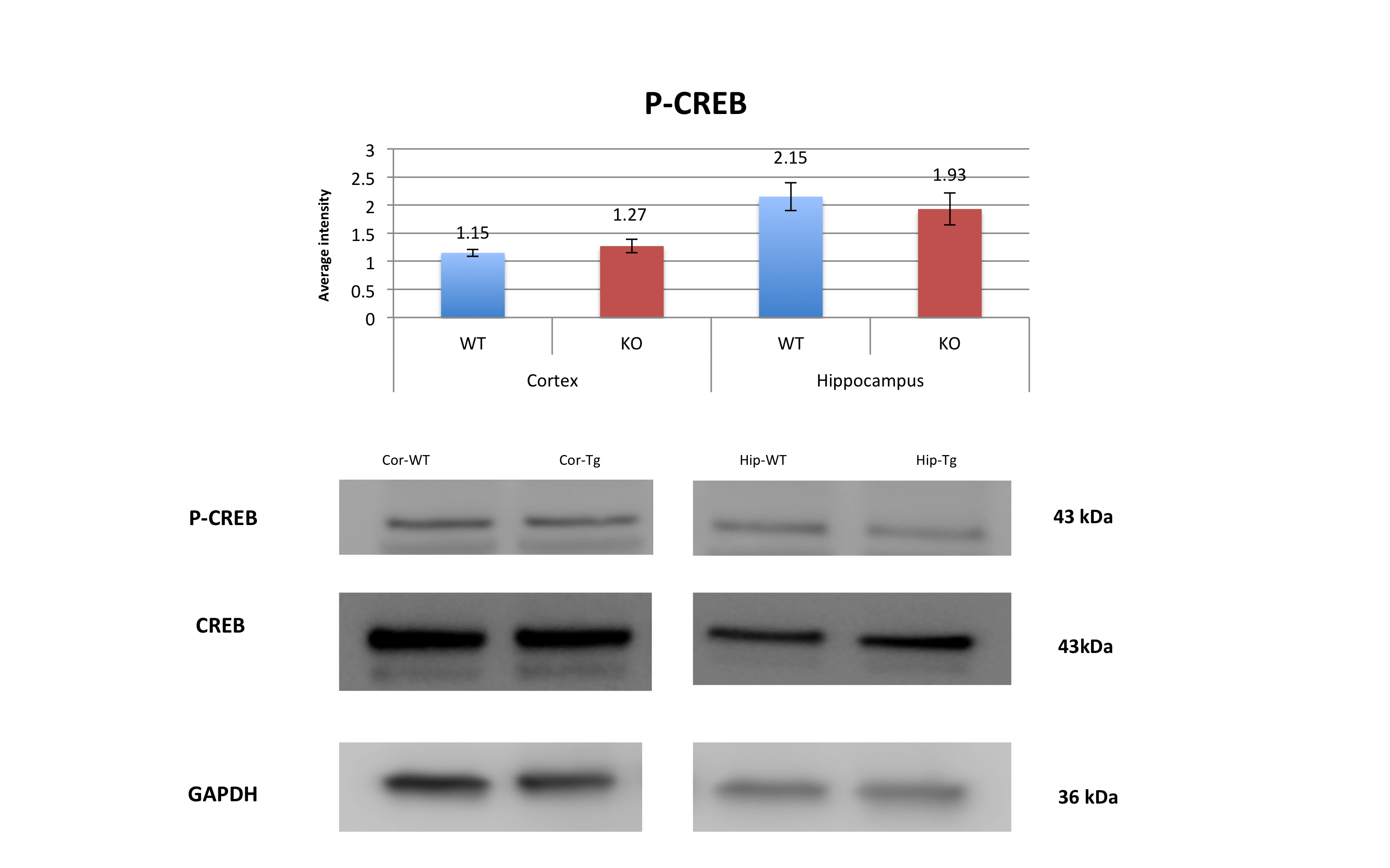

Supplement: Supplementary file 9 — Supplementary Figure 8 [file 41398_2019_388_MOESM9_ESM.tif]

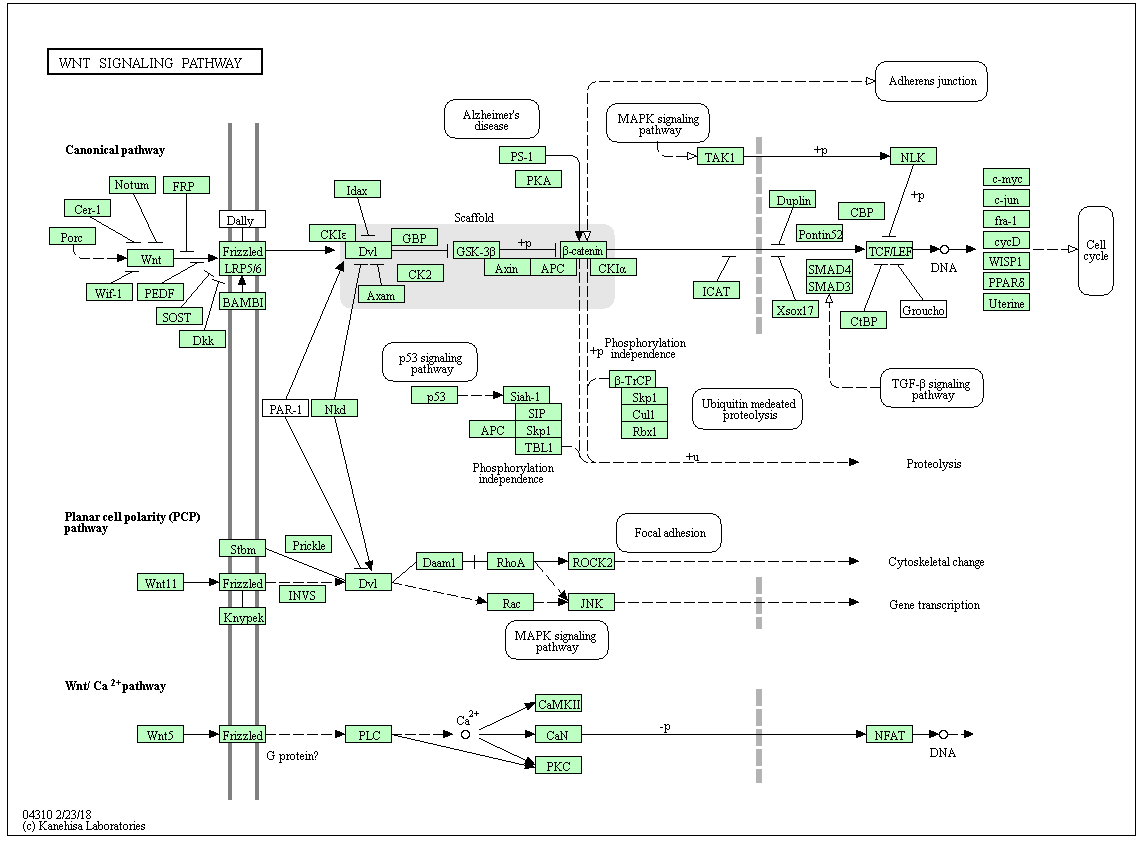

Supplement: Supplementary file 10 — Supplementary Figure 9 [file 41398_2019_388_MOESM10_ESM.png]
